# H1N1 influenza virus infection results in adverse pregnancy outcomes by disrupting tissue-specific hormonal regulation

**DOI:** 10.1371/journal.ppat.1006757

**Published:** 2017-11-27

**Authors:** Elizabeth Q. Littauer, E. Stein Esser, Olivia Q. Antao, Elena V. Vassilieva, Richard W. Compans, Ioanna Skountzou

**Affiliations:** Department of Microbiology & Immunology and Emory Vaccine Center, Emory University School of Medicine, Atlanta, Georgia, United States of America; St Judes childrens hospital, UNITED STATES

## Abstract

Increased susceptibility to influenza virus infection during pregnancy has been attributed to immunological changes occurring before and during gestation in order to “tolerate” the developing fetus. These systemic changes are most often characterized by a suppression of cell-mediated immunity and elevation of humoral immune responses referred to as the Th1-Th2 shift. However, the underlying mechanisms which increase pregnant mothers’ risk following influenza virus infection have not been fully elucidated. We used pregnant BALB/c mice during mid- to late gestation to determine the impact of a sub-lethal infection with A/Brisbane/59/07 H1N1 seasonal influenza virus on completion of gestation. Maternal and fetal health status was closely monitored and compared to infected non-pregnant mice. Severity of infection during pregnancy was correlated with premature rupture of amniotic membranes (PROM), fetal survival and body weight at birth, lung viral load and degree of systemic and tissue inflammation mediated by innate and adaptive immune responses. Here we report that influenza virus infection resulted in dysregulation of inflammatory responses that led to pre-term labor, impairment of fetal growth, increased fetal mortality and maternal morbidity. We observed significant compartment-specific immune responses correlated with changes in hormonal synthesis and regulation. Dysregulation of progesterone, COX-2, PGE2 and PGF2α expression in infected pregnant mice was accompanied by significant remodeling of placental architecture and upregulation of MMP-9 early after infection. Collectively these findings demonstrate the potential of a seasonal influenza virus to initiate a powerful pro-abortive mechanism with adverse outcomes in fetal health.

## Author summary

Maternal immunology is finely balanced to maintain a tolerant and supportive molecular environment for the developing fetus while continuing surveillance against foreign microbial threats. Influenza viral infection during pregnancy is a significant clinical risk for mothers and their newborns, increasing hospitalization, preterm birth, low birth weight, and maternal and neonatal deaths worldwide. In a mouse pregnancy model, we show how influenza virus infection disrupts the delicate and interconnected cytokine and hormonal signaling pathways that respond to respiratory pathogens. The health of mothers and offspring was impacted in our study, after pregnant mothers’ lung and placental architecture was compromised by infection. Influenza virus infection increased the stress on the mother’s body already present due to pregnancy, or reversed the hormonal environment required to establish and maintain healthy pregnancy. By dissecting the effects of inflammation post-infection throughout the mother’s anatomy, we can tailor anti-inflammatory treatments for the pregnant population. Also, thorough knowledge of immune responses will assist in tailoring vaccine design and dosage for this delicate period of women’s immunological and reproductive health.

## Introduction

Influenza virus has been responsible for four pandemics in the past century, with an additional global health burden of seasonal influenza-related illness estimated at five million cases of severe illness and nearly 500,000 deaths annually [1].

Pregnant women are among the high-risk groups who are more susceptible to seasonal and pandemic influenza viral infections, with pronounced lung immunopathology [2] and increased incidence of complications, such as pre-eclampsia, pneumonia or heart failure during all 3 trimesters of gestation resulting in high hospitalization rates and mortality [1, 3–8]. Notably, during the 2009 H1N1 pandemic, pregnant women in the United States showed a disproportionately high mortality rate, accounting for 5% of deaths while representing only 1% of the total population [9]. The majority of pregnant women who died of influenza-related illness during the pandemic were infected in the second and third trimesters of pregnancy [10].

Both seasonal and pandemic influenza virus have a substantial impact on the developing fetus. Infection during late-second or third trimester of pregnancy is associated with significant increases in miscarriages, stillbirths, and early neonatal diseases and death [1, 11]. Several studies indicate that infants born to influenza virus-infected mothers have an increased risk of developing health problems later in life ranging from chronic immune diseases to schizophrenia [12, 13].

Though the mechanisms predisposing pregnant women and their fetuses to an increased rate and severity of influenza virus infection-related complications are not fully elucidated, it is generally accepted that the feto-placental tolerance developed during pregnancy to support life of a semi-allogeneic fetus is a contributing factor to adverse outcomes [6, 7, 14, 15]. Dynamic immunological processes that occur throughout pregnancy are regulated by pregnancy hormones, whose receptors are found on most immune cells [7, 16–20]. Researchers have demonstrated a shift away from type Th1 cell-mediated responses toward enhanced type Th2 humoral-mediated responses during pregnancy, suggesting a protective role for the developing fetus, although these alterations could predispose to increased susceptibility to infections from respiratory viruses such as influenza [18, 21]. Inflammation plays a major role in tissue pathogenesis and it is more pronounced during the last 2 trimesters of pregnancy due to changes in cytokine levels that regulate fetal-placental tolerance, that suppress some cytokines (e.g. IFN-γ and VEGF), while elevating the levels of others (e.g. TNF-α and G-CSF) [14, 22, 23].

Presently there are very few studies that have examined the adverse effects of influenza virus infection in pregnancy mainly using the pregnant mouse model due to histological similarities of rodent to human placenta [24]. These reports demonstrated adverse effects of the 2009 pandemic influenza A (H1N1) virus on lung immunopathology [25–27], and more recently Kim et al reported severe pathogenesis of influenza B virus in the pregnant mouse model. However, the impact of seasonal H1N1 influenza A virus infection on maternal and fetal health during pregnancy, and the exact mechanisms leading to premature rupture of membranes (PROM) and preterm birth after influenza virus infection during pregnancy have not been fully elucidated [6, 11].

The objective of this study was to recapitulate the adverse outcomes of seasonal influenza virus infection in maternal, placental and fetal compartment using the mouse model. We aimed to correlate disease burden with progesterone and prostaglandin levels since their role is critical in lung and placental health; and assess the magnitude of endocrine and immunological changes that through tissue inflammation and pathology lead to maternal and fetal distress. To achieve this objective, we chose as a model influenza virus the seasonal H1N1 A/Brisbane/59/07 strain to sub-lethally infect BALB/c mice at the second third of gestation period and monitor maternal-fetal health. All correlations between progesterone and prostaglandins with compartment-specific inflammation and immune responses as well as tissue remodeling took place at 4 days post-infection, when weight loss was first exhibited in our mouse model and at which time point pregnant women are most susceptible to ICU admission and death in previous pandemics [10].

## Results

### Influenza viral infection during pregnancy results in shorter gestation period

**Fig 1:**
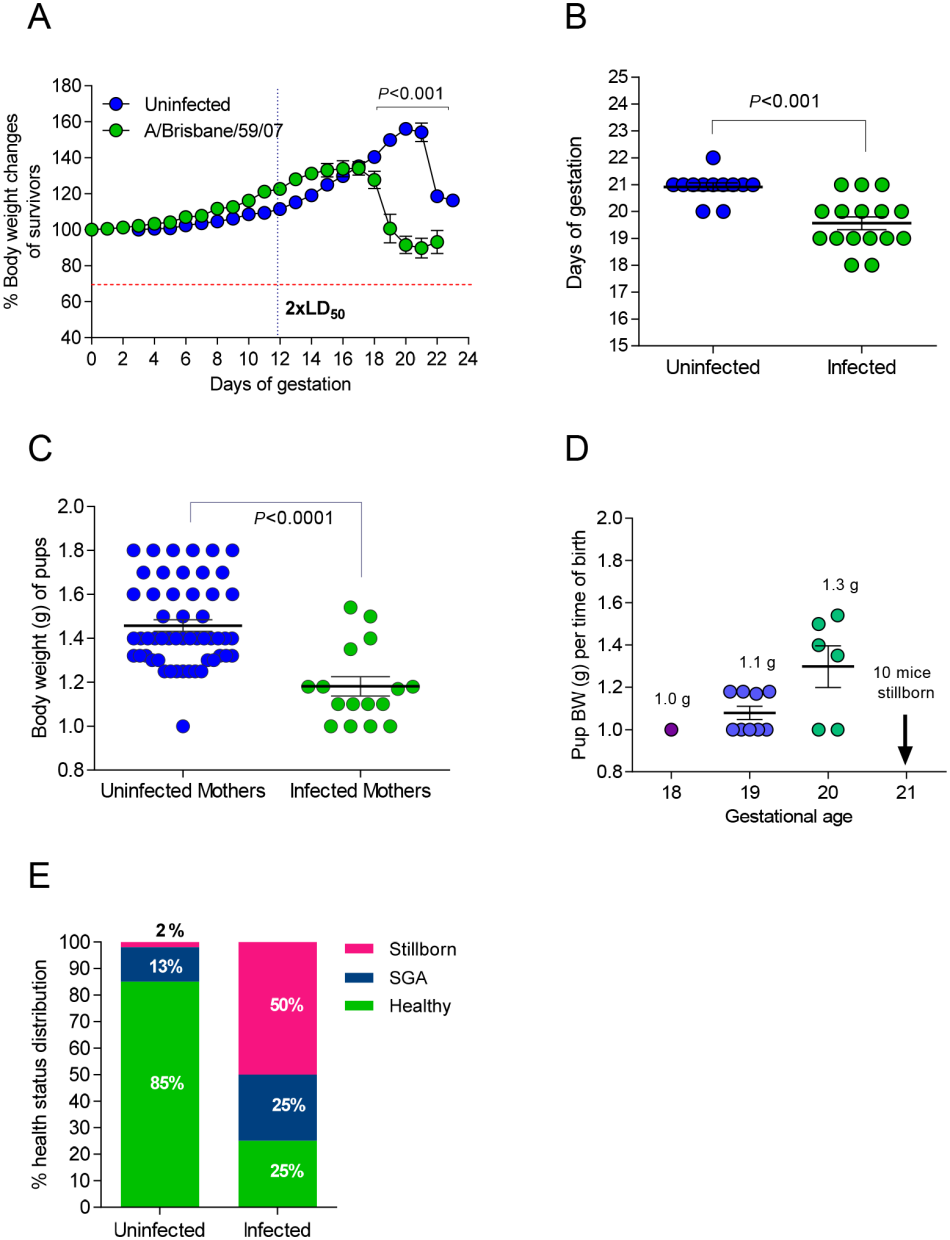
Influenza infection inhibits gestational development and offspring health. (A) Body weight (BW) fluctuations of pregnant BALB/c mice were tracked over the course of gestation. At day 12 of gestation (dashed vertical line) a cohort of pregnant mice (N = 6) was infected with 2xLD_50_ A/Brisbane/59/07. Weight changes were compared to uninfected pregnant controls (N = 12). *P*-values were calculated by two-way ANOVA. Error bars represent Standard Error of Mean (SEM). (B) Mean gestational length for infected (N = 13) and uninfected (N = 9) groups was calculated based on the dates of successful copulation and parturition of offspring. *P*-value was calculated by Student’s t-test. (C) The body weights (BW) of pups born to infected (N = 16 to 6 mothers) and uninfected mothers (N = 53 to 12 mothers) were recorded upon delivery. (D) The BW distribution of pups was categorized based on gestational age. Pups less than 1.25g were designated SGA. Pups greater than 1.25g were considered healthy. Average BW and SEM were plotted and *P* = value was calculated by Student’s t-test for C and D. (E) Percent distribution of health status of pups born to uninfected and infected mothers.

In order to assess the impact of seasonal influenza virus infection in maternal and fetal health, we first infected pregnant mice at day 12 of gestation and non-pregnant controls with a low dose of H1N1 A/Brisbane/59/07. With this approach we aimed to recapitulate human infection at the end of 2^nd^ to 3^d^ trimester of pregnancy with a low pathogenicity seasonal influenza virus. Uninfected pregnant controls reached a peak bodyweight that was 56.2% greater than that of their pre-pregnancy bodyweight (Fig 1A). The infected pregnant animals reached a peak bodyweight of 34% of their original bodyweight and the average gestation period decreased by 6.7% to 19.6 days (p<0.001) (Fig 1B). Additionally, pregnant mice infected during gestation dropped to 89% of their initial body weight following pre-term delivery, while uninfected pregnant mice remained at 116% of their initial body weight following delivery. We found that infection with a low pathogenicity influenza virus interrupted the normal progression of pregnancy to completion inducing pre-term labor in our mouse model. The initial experiment recapitulated the clinical phenotype of pre-term birth observed in human women infected mid-gestation with seasonal influenza virus [28].

### Mice born from influenza virus-infected mothers have an increased likelihood of stillbirth or being small for gestational age (SGA)

Next, we examined the impact of infection on offspring viability and health status of pregnant mice. Numbers of viable and non-viable pups were recorded, bodyweights were taken at birth and classified as non-viable (≤1 g), SGA (1.1–1.25 g) and healthy (>1.25 g) and monitored daily for growth curves. Litter size averaged 4.5 pups per uninfected healthy pregnant controls while infected mothers gave birth to an average of 2.7 pups per litter; however, because infected mothers often consume sick or stillborn offspring, these results are not necessarily reflective of number of offspring carried to parturition. The average weight of offspring from uninfected pregnant mice was 1.46g. When mothers were infected with the virus, the body weight of newborns was 19% lower, averaging 1.18g (p<0.0001) (Fig 1C). Pups born from infected mothers at days 18 and 19 of gestation had lower body weight compared to the pups born at day 20; however, 10 mice born on day 21 were all stillborn (Fig 1D). Thus, the length of pregnancy did not necessarily correlate with the health outcome of the offspring; that is, SGA condition was not dependent on the length of gestation. In uninfected pregnant mothers, 85% of pups were born with a healthy weight, while 13% were SGA pups and 2% were stillborn (Fig 1E). Infection with seasonal influenza virus reduced the number of healthy offspring to 25%; the remaining pups were 25% SGA, and 50% stillborn (Fig 1E). Overall, offspring born from infected mothers were approximately 20% smaller in terms of body weight compared to offspring of uninfected mothers. Infection nearly doubled the incidence of SGA pups from 13% to 25%, and the likelihood for stillbirth increased by 35-fold from 2% to 70% following infection (Fig 1C, 1D and 1E). Low birthweight or SGA offspring are a common outcome of pregnant mothers infected with pandemic H1N1 influenza virus during the second and third trimester and has been observed in cohorts of pregnant women infected with seasonal H1N1 influenza virus in Nova Scotia [6]. Thus our model replicates more serious clinical birth complications associated with influenza A viral infection during mid- to late-gestation pregnancy.

### Pregnancy reduces viral clearance in the lungs of influenza virus-infected mice

**Fig 2:**
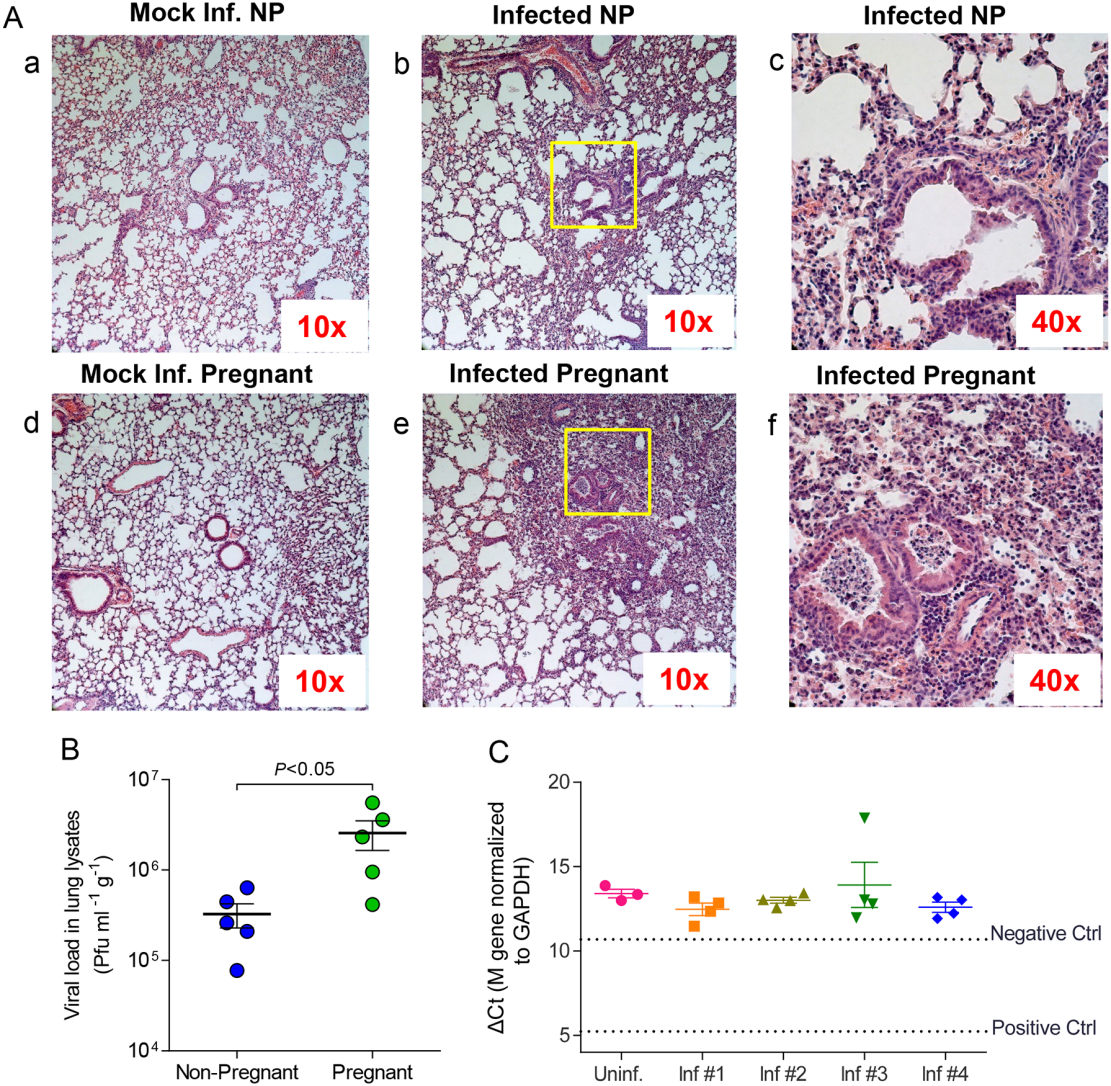
Pregnancy results in increased inflammation in the lungs and enhanced viral growth in lungs but not detected in placenta. A. Lungs from mock infected (a, d) and infected (b, c, e, f) pregnant and non-pregnant mice were stained with hematoxylin and eosin (H&E) 4 d.p.i. with mouse-adapted A/Brisbane/59/07 or mouse lung lysate at an equivalent dilution. (b, e) Lung sections at 10x magnification; (c, f) Inset boxes in (b, e) at 40x magnification represent lung structures. B. Viral load was quantified from lung lysates (N = 5 per group) 4 d.p.i via plaque assay in MDCK-derived cell lines and normalized per gram of tissue weight. C. Viral RNA was isolated from placental lysates (N = 4–5 per group) and probed for the presence of M gene RNA (A/Brisbane/59/07) via qPCR. Influenza-virus specific RNA was not detected below the negative control threshold (RNA isolated from mock-infected BALB/c lungs) or positive control threshold (RNA isolated from infected BALB/c lungs). Inf.: Infected mice; NP: Non-pregnant mice.

The severity of viral infection in pregnancy was initially assessed by examination of various organs for systemic spread of pathogen, viral load and tissue inflammation at 4 d.p.i. The results from lungs of infected pregnant mice were compared to those of non-pregnant infected controls. Although both pregnant and non-pregnant infected lungs were heavily infiltrated by neutrophils showing intense inflammation (Fig 2A), viral loads were 8-fold higher (p<0.05) in pregnant animals compared to non-pregnant ones, suggesting hampered virus clearance during pregnancy (Fig 2B). Interestingly, lungs of mock-infected pregnant mice showed increased airway inflammation over mock-infected non-pregnant mice, indicating that there is increased physiological stress in the lungs during pregnancy (Fig 2a: a, d).

Viral titers were undetectable in the placentas and fetuses of infected mice. This was further confirmed with qPCR measuring viral RNA (M gene) levels (Fig 2C). Lack of detectable viral RNA in placental and fetal tissues indicates that transplacental transmission of influenza virus from mother to child is unlikely and that retardation in fetal development was not due to *in utero* infection. These findings correspond with clinical reports on pregnant mothers infected with influenza A virus during pregnancy; while several H1N1 strains can infect fetal trophoblast cells *in vitro*, clinical evidence of vertical transmission is uncommon and inconclusive [29–31]. Despite the presence of influenza virus receptors α-2,6 sialic acids on human placental membranes, only H5N1 viruses have been reported to transmit via maternal vertical transmission [32, 33]. This indicates that the negative effects on offspring born to mothers infected with influenza virus during pregnancy are mediated not by direct infection of placenta and fetus, but indirect causes such as dysregulated hormonal signaling, increased inflammation, or immune system activation against placental and/or fetal tissue.

### Progesterone and PGF2α regulate compartment-specific cytokine signaling during gestation in the absence of infection

**Fig 3:**
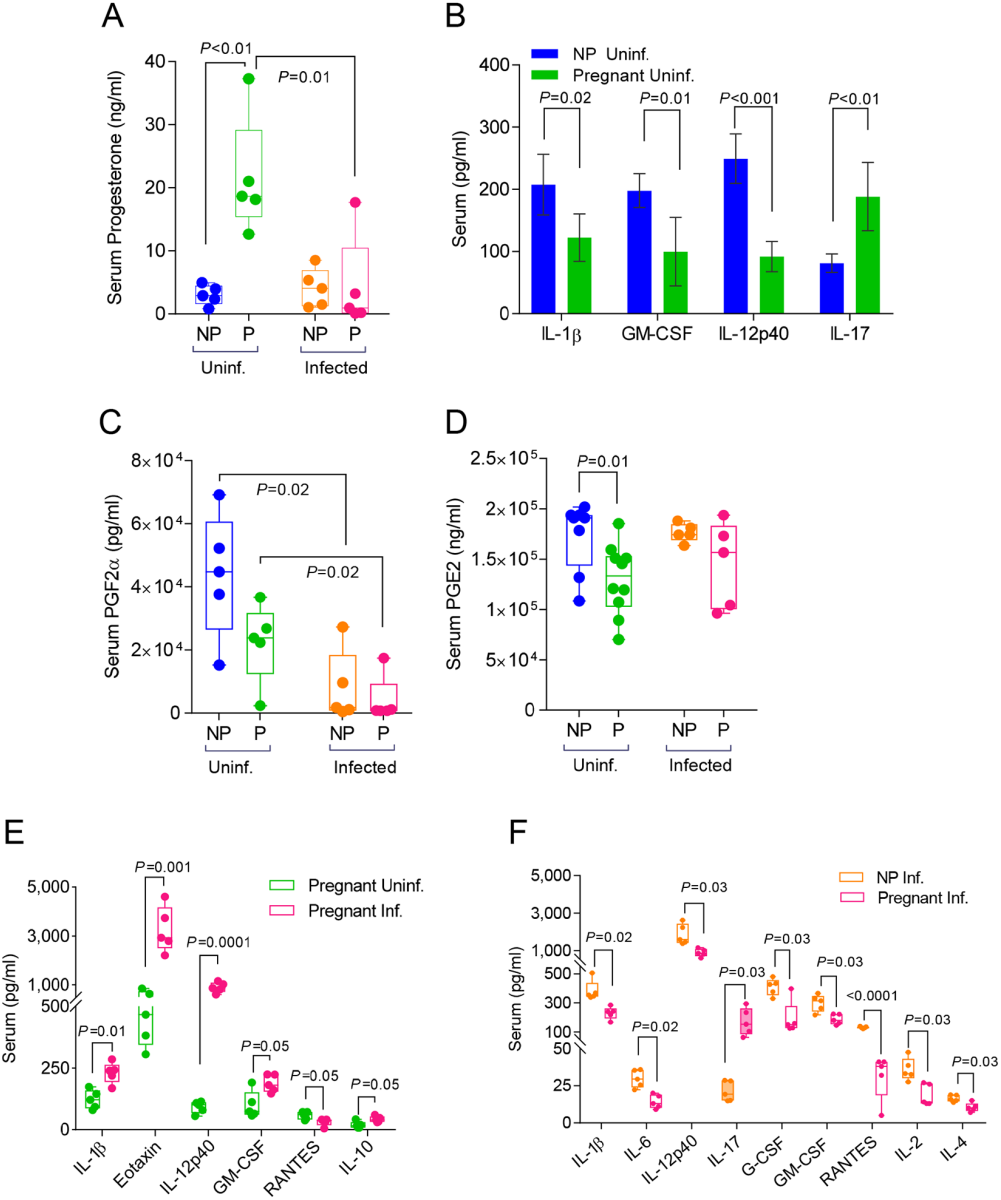
Viral infection disproportionately reduces cytokine and hormone expression in the sera and lungs during pregnancy. Hormone expression in sera was quantified via ELISA in uninfected and infected non-pregnant mice for A. progesterone; C. prostaglandin F2α (PGF2α); and D. prostaglandin E2 (PGE2). Cytokine and chemokine expression in sera was quantified via Bio-Rad 23-plex assay in B. uninfected pregnant (E16) (N = 5) and non-pregnant mice (N = 5); E. uninfected and infected pregnant (E16; 4 d.p.i) (N = 5 per group); and F. infected (4 d.p.i) non-pregnant and pregnant (E16) mice (N = 5 per group). Student’s t-test was performed between selected groups and significance noted above asterisk brackets. Inf.: infected; Uninf: uninfected; NP: non-pregnant; P: pregnant mice.

Progesterone promotes endometrium and uterine changes to support embryo implantation and feto-placental development. Insufficient progesterone concentrations can lead to preterm delivery and miscarriage in humans and rodents [34, 35]. To assess the effect of influenza virus infection on systemic progesterone levels and subsequently in pre-term labor, we first measured cytokine expression and progesterone levels in serum of uninfected pregnant and non-pregnant mice (Fig 3).

Serum progesterone was 7-fold higher (p<0.01) in uninfected pregnant mice compared to non-pregnant controls as is expected during healthy gestation (Fig 3A). These levels inversely correlated with the levels of pro-inflammatory cytokines IL-1β, IL-12p40 and GM-CSF showing a reduction of 41%, 50% and 63% respectively when compared to their levels in non-pregnant mice (p=.01) (Fig 3B, S1 Table). None of the Th2 (IL-3, IL-4, IL-5, IL-10, IL-13) or Th1 (IL-2, IL-12p70, IFN-γ) cytokines showed any differences between pregnant and non-pregnant mice (S1 Table).

To further establish the threshold of inflammation in the pregnant mouse model, we measured the levels of serum prostaglandin F2α (PGF2α) and prostaglandin E2 (PGE2) in uninfected pregnant mice and compared them to uninfected non-pregnant controls. PGE2 selectively suppresses effector functions of macrophages and neutrophils and the Th1, CTL and NK cell-mediated type-1 immunity, but promotes Th2, Th17, and T_reg_ responses [36]. In contrast to increased levels of progesterone during pregnancy, PGF2α and PGE2 were reduced by 50% and 33% respectively in serum of pregnant mice compared to non-pregnant controls (Fig 3C and 3D). Thus we established in our mouse model that pregnancy hormones or hormone-like mediators of homeostasis are subjected to systemic changes to prepare an immune environment trending toward anti-inflammatory responses.

### Infected pregnant mice show lower levels of serum inflammatory markers than non-pregnant controls

Hormonal and cytokine responses were further confounded during pregnancy following influenza virus infection. Infection caused a 5-fold reduction of serum progesterone in pregnant mice 4 d.p.i. to levels similar to those seen in non-pregnant mice (Fig 3A). There was a significant inverse correlation between serum progesterone levels and lung viral load (r^2^ = 0.8, p = 0.04) (S1C Fig). Similarly, serum PGF2α levels were approximately 5 times lower than pre-infection in both pregnant and non-pregnant cohorts (p = 0.02) (Fig 3C) whereas PGE2 did not show any significant differences (Fig 3D).

Infection in pregnancy elevated the serum inflammatory cytokines IL-1β, IL-12p40, GM-CSF and eotaxin from 2 to 10 times whereas it significantly reduced RANTES (Fig 3E; S1 Table). When compared to infected non-pregnant controls, pregnant mice had less than half the number of upregulated inflammatory cytokines (IL-1β, IL-6, IL-12p40, eotaxin, G-CSF, GM-CSF, MIP-1α, TNF-α, RANTES,) (S1 Table). The majority of these cytokines had approximately 50% lower levels in pregnant mice (Fig 3F). Interestingly, IL-10 increased significantly only in infected pregnant mice (p = 0.02) suggesting a robust regulatory response to infection (Fig 3E). Pro-inflammatory IL-17 which is detected mainly in serum and placenta [37] was elevated 2-fold during pregnancy (Fig 3B and 3F). Although IL-17 production was not affected by virus infection, its levels were significantly higher than those observed in infected non-pregnant animals (Fig 3F, S1 Table). These data show that pregnancy results in a unique signature of systemic antiviral cytokine expression. Progesterone and PGF2α reduction led to an inflammatory response although its magnitude was lower than the response seen in the control group.

### Pregnancy increases inflammation in the lungs following H1N1 influenza virus infection

Progesterone increase in pregnancy results in relaxation of smooth muscles in the airways, dilating the bronchial tissue [38]. PGE2 and PGF2α are key players in lung’s physiological response to infection and airway reactivity [38]. PGE2 is a vasodilator that increases the permeability of the lung vasculature [39, 40] and works with PGF2α, a bronchoconstrictive hormone to finely tune lung function [41]. Influenza virus infection damages lung epithelial cells, releasing free reactive oxygen species (ROS) that activate cyclooxeganse-2 (COX-2) via the arachidonic acid pathway and upregulate synthesis of PGE2 [42]. In order to assess the effect of these hormones on inflammatory responses in infected lungs of pregnant mice, we determined their levels along with a panel of 23 cytokines and chemokines in tissue lysates of pregnant and non-pregnant mice 4 d.p.i.

**Fig 4:**
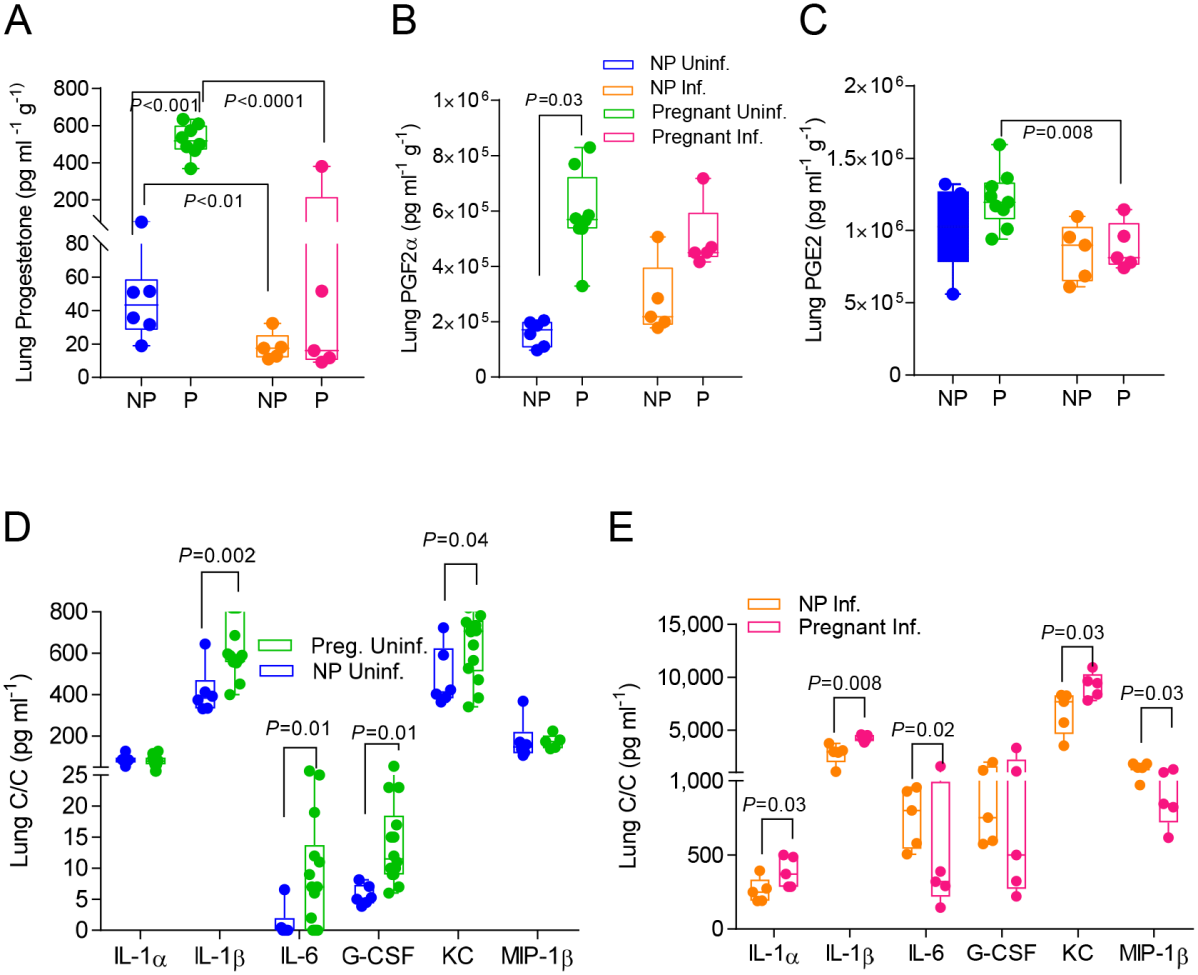
Pregnancy increases lung tissue expression of proinflammatory cytokines and chemokines while dampening the upregulation of progesterone and PGF2α following infection. Hormone expression in lung lysates was quantified via ELISA in uninfected (N = 6) and infected (N = 5) non-pregnant and pregnant mice for A. progesterone; B. prostaglandin F2α (PGF2α); and C. prostaglandin E2 (PGE2). Cytokine and chemokine expression in sera was quantified in lung lysates of D. uninfected pregnant (E16) (N = 8) and non-pregnant mice (N = 6); E. infected pregnant (4 d.p.i., E16) (N = 5) and non-pregnant mice (N = 5). Student’s t-test was performed between selected groups and significance noted above asterisk brackets. Abbreviations for groups are as described in Fig 3.

Pregnancy increased expression of lung progesterone 11.6-fold (p<0.001) and PGF2α 2.7-fold (p = 0.03) (Fig 4A and 4B), while no detectable changes were found in PGE2 levels between pregnant and non-pregnant mice (Fig 4C). Notably, in the absence of infection, pregnant uninfected mice overexpressed lung neutrophil-recruiting chemokines (KC, 1.37 times higher) and cytokines (IL-1β, IL-6 and G-CSF; 1.64-, 7.6- and 3-times higher respectively) relative to non-pregnant controls (Fig 4D, S2 Table) [43]. These data suggest that these changes may prepare the lung environment during pregnancy to fight microbial insults, more so than during a non-pregnant state.

Infection reduced progesterone expression in the lungs of pregnant animals by 5-fold (p<0.0001) when compared to non-pregnant mice that showed a 2-fold reduction (p = 0.01) (Fig 4A). Moreover we observed a strong correlation between lung progesterone with viral load (r^2^ = 0.79, p = 0<0.0001) (S1D Fig). Following the same trend, PGE2 levels when significantly decreased (p = 0.008) (Fig 4C). However, PGF2α lung expression was not altered after infection in either pregnant or non-pregnant mice (Fig 4B).

While influenza virus infection caused dramatic increases in expression of inflammatory cytokines and chemokines in lungs of both pregnant and non-pregnant mice (S2 Table), we detected a few differences between these groups. The levels of IL-1α, IL-1β and KC were 50%, 200% and 40% higher than those estimated in infected non-pregnant controls, while IL-6 levels were 50% lower in the pregnant group (Fig 4E, S2 Table). The results on IL-1α and IL-6 are in agreement with previous reports on the role of IL-1α as a negative regulator of IL-6 expression [44]. Influenza virus infection upregulated MIP-1α expression in both pregnant and non-pregnant cohorts reaching similar levels (S2 Table) whereas MIP-1β expression was 40% lower in pregnant animals (Fig 4E, S2 Table). While both cytokines are involved in cellular recruitment and inflammation at the site of infection, they signal through different receptors. MIP-1α signals through CCR1, CCR4, and CCR5 and MIP-1β signals directly through the CCR5 [45], suggesting that pregnancy may induce different pathways signaling in response to infection.

Our findings point to a model for the excessive morbidity seen in infected pregnant mice. Pregnancy induces a combination of physiological changes, including vasoconstriction in the lungs via PGF2α, the negative impact of infection on progesterone and PGE2 expression, and robust inflammatory responses in the lungs. Decreased bronchodilation and intense neutrophil infiltration in the alveolar spaces leads to respiratory failure, which is evident also in lung histopathology (Fig 2A) [46–48].

### Infection upregulates labor-inducing hormones and inflammatory cytokines in the placenta

**Fig 5:**
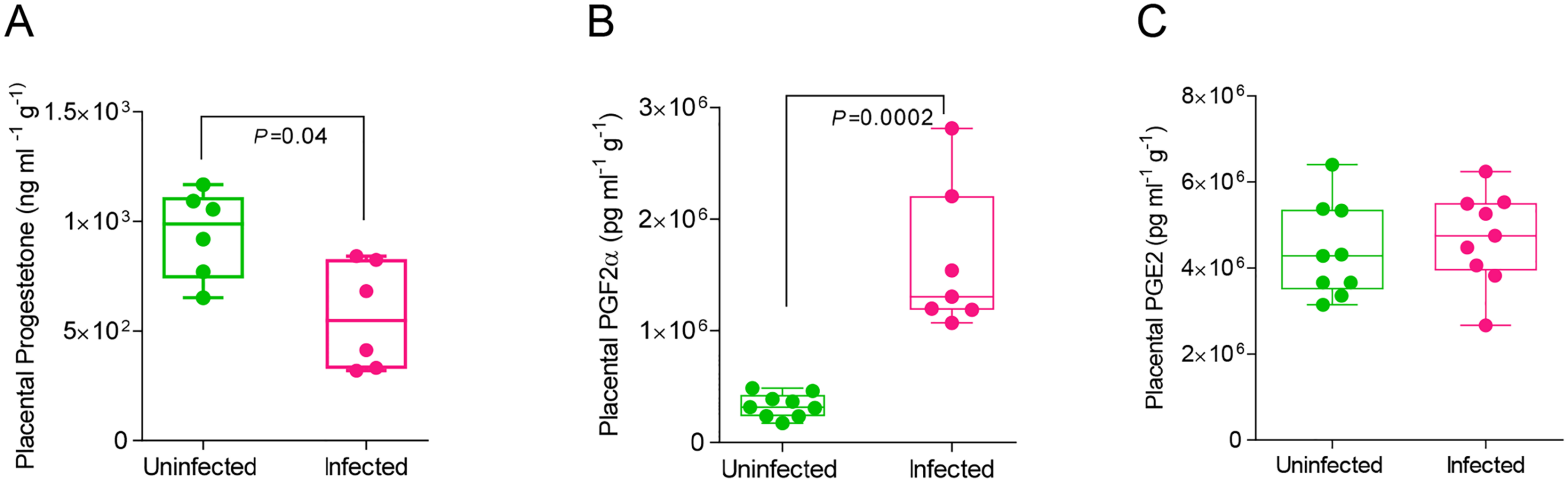
Infection increases placental expression of contraction-inducing PGF2α and reduces pregnancy-supportive progesterone. Placental lysates were isolated from uninfected and infected pregnant mice (E16). Hormone expression was quantified via ELISA for A. progesterone (N = 6 per group); B. prostaglandin F2α (PGF2α) (N = 9, uninfected, N = 7, infected) and C. prostaglandin E2 (PGE2) (N = 9 per group). Student’s t-test was performed between selected groups and significance noted above asterisk brackets.

In addition to their effects on lung architecture and immune responses, progesterone, PGE2 and PGF2α were measured following infection of pregnant mice because of their respective roles in uterine contractility. Progesterone elevation in pregnancy supports fetal development by thickening the endometrium to allow for embryo implantation and suppressing inflammation in the uterus. PGE2 increases vasodilation, induces uterine contractions, and decreases T-cell proliferation and lymphocyte migration [49] while PGF2α initiates vasoconstriction of uterine and endometrial blood vessels [50], resulting in induction of labor [6, 12, 13, 51–53]. At the same time, PGF2α stimulates the production of pro-inflammatory cytokines and may enhance uterine production of leukotriene B4 (LTB4) that in turn activates various neutrophil functions [54]. We found that progesterone levels in the placentas of infected pregnant mice were decreased by 40% compared to uninfected pregnant mice (p=.04) (Fig 5A). In contrast, PGF2α levels were almost 5-fold higher in the same group compared to uninfected controls (p=.0002) (Fig 5B). Placental lysates did not show a difference in PGE2 expression (Fig 5C) after infection.

Progesterone and PGF2α expression were inversely correlated with viral load following infection although significant association between measured parameters was only observed in progesterone (r^2^ = 0.46, p = 0.007 and r^2^ = 0.44, p = 0.22 respectively) (S1B and S1E Fig). The association of serum, lung or placental progesterone with viral load supports the notion that seasonal influenza virus infection during gestation disrupts the production of progesterone, and provides evidence an explanation for early termination of pregnancy and spontaneous abortions.

Fold changes of cytokine expression in the placentas and fetuses of pregnant mice were also compared before and after infection. Eleven placental cytokines showed suppressed expression ranging from 29–72% after infection (S2 Fig, S3 Table). G-CSF and RANTES however were enhanced by 4.8- and 1.5-fold respectively. Similarly, fetuses showed a dramatic decrease in immune responses, although pre-infection cytokine and chemokine levels were very low compared to the placental compartment. With the exception of IL-1α, which was increased following infection, cytokine expression was decreased in the fetus post-infection by at least 60% (S2 Fig, S3 Table). This suppression indicates that the placenta and fetus are effectively shielded from the inflammatory cytokine signature of the mother’s circulatory system and that the physiological changes induced by progesterone and prostaglandins are likely causes of poor fetal and offspring outcome.

### Infection disrupts placental architecture via inflammation and protein degradation

**Fig 6:**
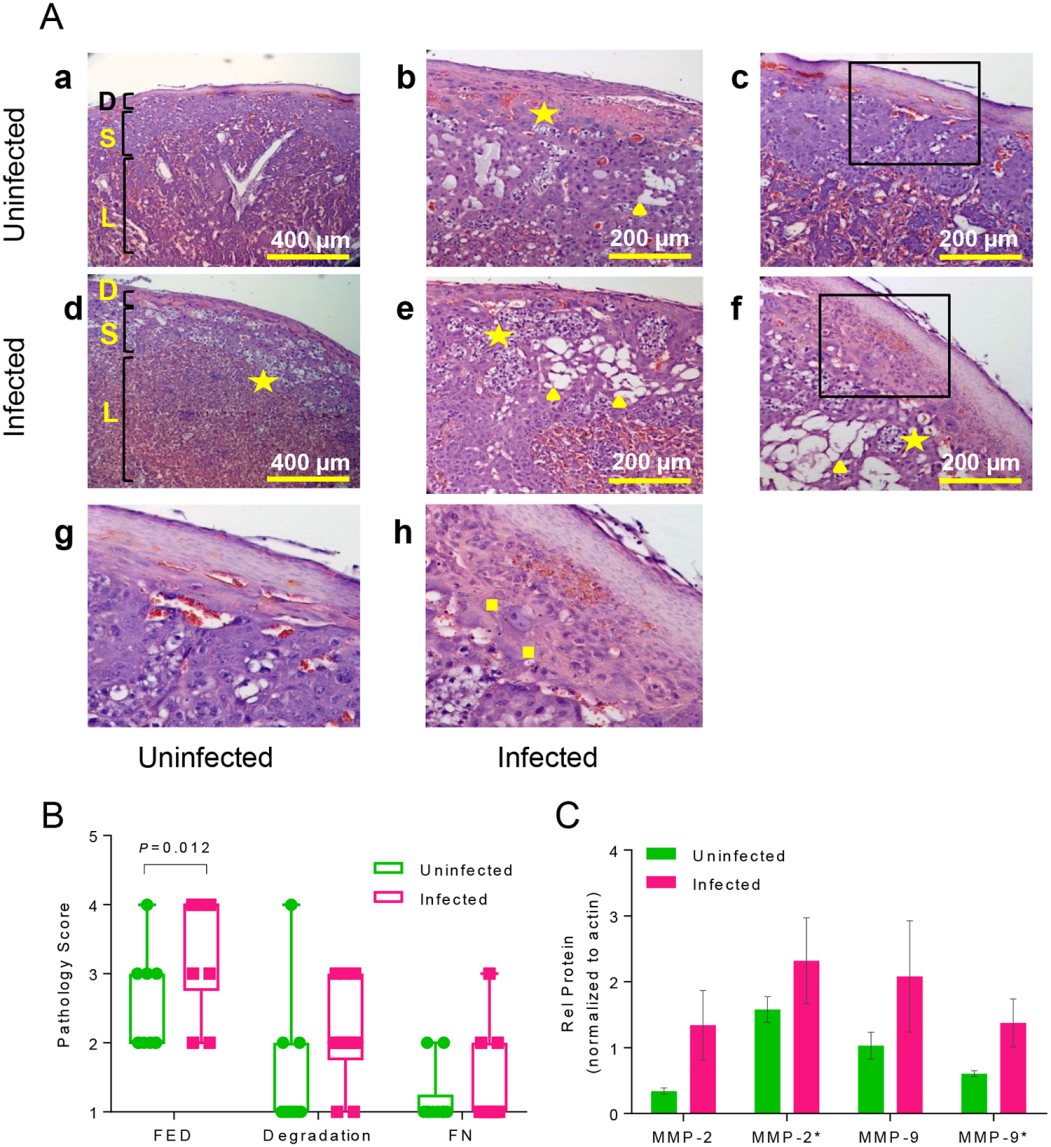
Infection damages placental architecture and increases activation of structural protein degrading matrix metalloproteinases (MMPs). A. Placentas from uninfected [**a, b, c, g**] (N = 8) and infected [**d, e, f, h**] (N = 8) pregnant mice (E16) were embedded in paraffin, sectioned in 4 μM, H&E stained and imaged with a Zeiss brightfield microscope. Infection increases gaps within the spongiotrophoblast layer (S) between the decidual layer (D) and the placental labyrinth (L) [**a, d**; **10x**]. Gaps are marked by fetal endothelial nuclei suspended in tissue [stars] and empty space [triangles] distinct from maternal blood sinuses and fetal blood vessels [**d, 10x; b, e, f, 20x**]. Fibrinoid necrosis [squares] beneath the decidual layer was increased in placentae from infected mice [**h, inset of box in f**] compared to uninfected mice [**g, inset of box in c**]. B. Histology slides were randomized prior to imaging and scored blindly for the incidence of fetal endothelial death (FED; N = 10, p = 0.012), degradation of the spongiotrophoblast layer (Degradation; N = 10, p>0.05), and fibrinoid necrosis (FN; N = 10, p>0.05). C. Protein expression of matrix metalloproteinases (MMP-2, MMP-9; active forms denoted with *) in placentas in uninfected and infected mice at E16 quantified by Western blot. Two-way ANOVA: protein maturation, p = 0.064, ns; infection, p = 0.007, **.

Placenta is the sole source of nutrients and oxygen to the developing fetus. The placenta in both humans and rodents is derived from the maternal endometrial decidua and fetal trophoblasts [55]. The development of this critical organ is carefully negotiated through regulation by pregnancy hormones, initiated by prolactin and progesterone, and tightly regulated immune tolerance by uterine regulatory T cells [56]. Inflammation in the uterus and placenta due to infection or autoimmune responses has been linked to preeclampsia, endometriosis, and spontaneous abortion [57, 58] yet influenza virus has not been definitively shown to cross the maternal-fetal barrier in mice or humans. Viral infection in the placenta was examined and yet no viral RNA (M gene) was detected via qPCR (Fig 2C). Hence, the effect of influenza infection on placental function and health was examined via histology and molecular assays. Placentas from uninfected mothers at E16 (16 days of gestation) (Fig 6A: a, b, c, g) and mothers 4 d.p.i. (Fig 6A: d, e, f, h) were compared. Placentas from uninfected mothers maintained structural integrity between the maternal decidual layer, the fetal spongiotrophoblast layer, and the placental labyrinth (Fig 6A: a) while placentas from infected mothers (Fig 6A: d) showed increased regions of fetal endothelial cellular death (**stars**) with dark-blue nuclei suspended within the tissue, indicating that infection increases cellular death within the placenta. Infection increased gaps within the spongiotrophoblast layer (▲), a region through which maternal spiral arteries cross to deliver oxygen to fetal blood within the labyrinth (Fig 6A: b, e, f). Infection also increased the incidence of fibrinoid necrosis in-between the maternal decidual layer and the fetal spongiotrophoblast (■) region. (Fig 6A: h) [59]. Histology slides were scored for the incidence of fetal endothelial cellular death (FED), degradation of the spongiotrophoblast layer, and fibrinoid necrosis (FN); infection increased the occurrence of these indicators of poor placental health (Fig 6B).

To determine underlying molecular causes of the placental spongiotrophoblast degradation, matrix metalloproteinase (MMP) expression was quantified via Western Blot in placental lysates from infected and uninfected pregnant mice at E16 (Fig 6C). MMPs are proteolytic enzymes, localized in the placenta, that remodel tissues throughout the body via endopeptidase activity towards extracellular membranes (ECM), breaking down tightly adherent cells within the tissue [60–62]. MMP-2 and MMP-9 are gelatinases that have been widely implicated in placental pathology and preterm labor, both in rodent models and in humans [57, 63–66]. MMP-9 can be induced by high levels of IL-1β via the p38 MAPK signaling pathway [61, 67, 68], consistent with the increased levels of IL-1β observed in placentas of infected mice (S3 Table). Infection increased placental expression of the pro-enzyme and activated enzyme form of MMP-2 and MMP-9 (Fig 6C), thus exposing both the placenta and the amniotic membranes surrounding the fetus and placenta to degradation.

### Infection alters the expression of immune-responsive hormone regulators COX-2 and PIBF

**Fig 7:**
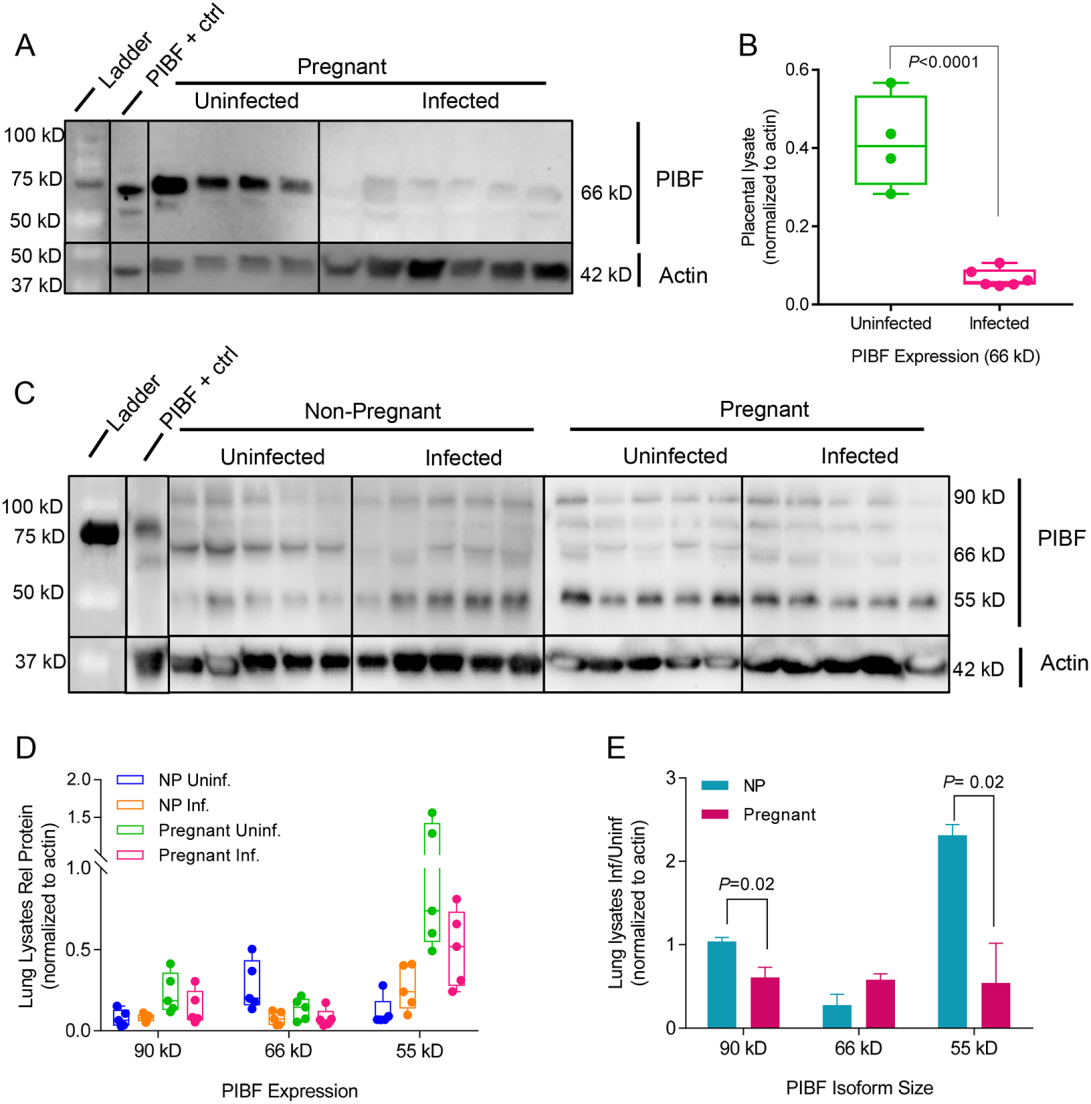
Infection changes pregnancy-supportive PIBF expression in the placenta and lungs. PIBF isoforms (90 kD, 66 kD, 55 kD) were detected using Western blots of A. placental and C. lung lysates. Protein expression is represented as isoform density normalized to loading control anti-beta actin (42 kD) for each sample. The effect of infection was measured by dividing the average density of bands from tissues from infected mice by the uninfected controls for both pregnant and non-pregnant mice. (B) Relative protein normalized to actin for uninfected (N = 4) and infected (N = 6) placental lysates. (D) Relative protein normalized to actin for lung lysates (N = 5 per group. (E) Ratio of infected vs uninfected lung lysates (normalized to actin). Uninf: uninfected; NP: Non-pregnant; Inf: Infected mice. P-values were determined by Student’s t-test.

Sex hormone synthesis and feedback pathways are intrinsically linked with innate immune signaling pathways of TNF-α, IL-1β, IL-4, IL-6, IL-10, IL-17, and IFN-γ [42, 69, 70]. Hence, we quantified hormone regulators, progesterone-induced blocking factor (PIBF) and COX-2 in order to examine the interdependent relationships in endocrine and innate signaling in our pregnancy model. Successful outcome of gestation requires expression of PIBF, a progesterone-responsive immunomodulatory protein with multiple active isoforms: 90kD, 66kD, and 55kD [71, 72]. PIBF promotes Th2 cytokine production [73] and inhibition of natural killer (NK) cell activity [34, 51]. While this function is crucial to limiting cytolytic activity in the uterus and placenta reduction of NK activity in lungs leaves the pregnant mother vulnerable to infection. Expression of PIBF isoform 66kD in the placenta was nearly abrogated following infection while 90 kD and 55 kD isoforms were undetectable (Fig 7A and 7B). In non-infected non-pregnant and pregnant mouse lungs, these isoforms were differentially expressed. The isoforms 90 kD and 55 kD showed 3-fold and 9-fold higher expression respectively in pregnant mice as compared to non-pregnant controls. In contrast, the levels of 66 kD isoform were 2-fold lower when comparing the pregnant to the non-pregnant group. Following infection of pregnant mice, isoform 66 kD was reduced 3-fold and isoform 55 kD was increased 2-fold. In infected pregnant mice, all isoforms were uniformly suppressed by 2-fold (Fig 7C, 7D and 7E). The results of this study demonstrate that different isoforms are highly expressed in placenta and lungs of pregnant mice, that infection down-regulates all isoforms and that there is a differential expression of isoforms in lungs during pregnancy. Notably the regulation of isoform 55 kD in pregnant and non-pregnant population follows opposite trends, suggesting a role in immunomodulation of placental-fetal interface.

**Fig 8:**
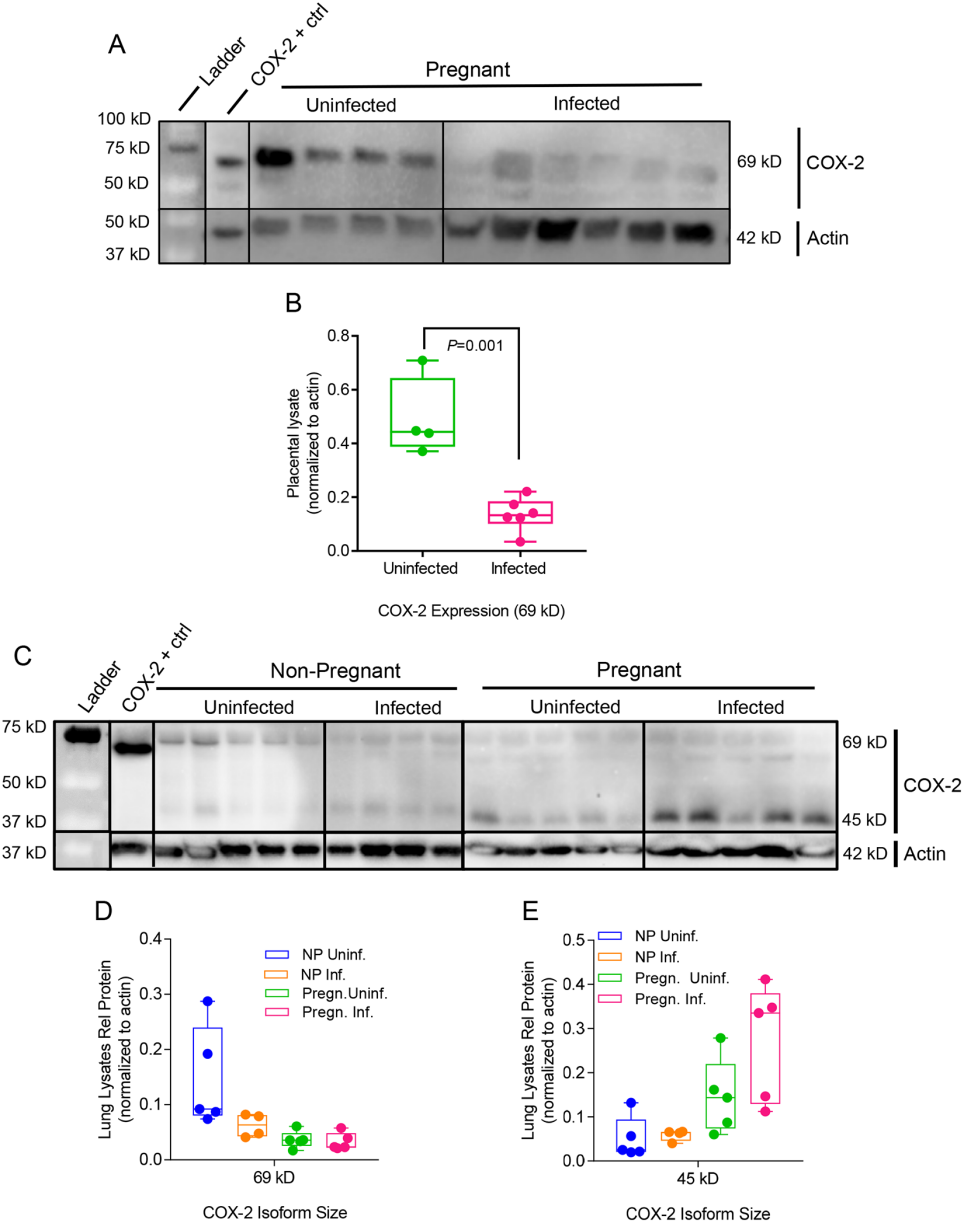
Infection and pregnancy increase COX-2 expression in the lungs. (A) Placental and (C) lung lysates collected 4 d.p.i were probed via Western blot for COX-2 expression. Protein expression is represented as isoform density normalized to loading control anti-beta actin (42 kD) for each sample. (B) COX-2 69 kD expression was the only detectable band in these placental lysates (Uninfected, N = 4; Infected, N = 6) and it was significantly lower in infected mice. COX-2 isoforms (D) 69 kD and (E) 45 kD were detected in lung lysates (N = 5 per each group except NP Infected mice; N = 4). Group annotations are as described in Fig 7. P-values were determined by Student’s t-test.

COX-2 is a key regulator in the arachidonic acid and prostaglandin synthesis pathway. Influenza virus infection induces COX-2 expression, thus increasing secretion of PGF2α in bronchial epithelial cells [42]. Previous studies demonstrated that COX-2 deficiencies reduce the recruitment of macrophages and neutrophils to the site of influenza viral infection, and COX-2^-/-^ mice infected with influenza virus had reduced lung viral clearance compared to wild-type infected mice [69] [74]. COX-2 placental expression was decreased upon infection (Fig 8A and 8B) which is consistent with lack of upregulation of PGE2 in the placenta following infection (Fig 5C). Thus, preterm birth in our model is correlated with the physiological changes mediated by the withdrawal of pregnancy-supportive progesterone, the increased expression of labor-inducing PGF2α, and upregulation of structure-damaging degradative proteinases in the placenta rather than increased inflammation mediated by COX-2, PGE2, or cytokines.

Pregnancy elevated expression of active COX-2 45kD in the lungs (Fig 8C and 8E). Infection did not exert a significant effect on active 45 kD COX-2 expression in the lungs of non-pregnant mice but resulted in a 2-fold increase during pregnancy (Fig 8E). Enhanced COX-2 expression during pregnancy may explain increased expression of neutrophil-recruiting chemokines in the lungs (Fig 4E), providing a regulatory mechanism for the enhanced immunopathology seen following infection.

## Discussion

Influenza viral infection disrupts the supportive molecular environment for fetal development and offspring health. The objective of this study was to investigate how seasonal H1N1 influenza virus infection affects the outcomes of pregnancy and how pregnancy alters the innate immune response during infection in a BALB/c mouse model. This study is unique in that it ties together hormonal changes induced by infection with cytokine dysregulation across three compartments; maternal, placental and fetal. We demonstrate that influenza virus infection alters key hormone levels required to maintain healthy pregnancy, and results in increased immunopathology thus compromising maternal and fetal health. Our study expands upon previously published work from Kim HM et al[27], Kim JC et al [75], Klein and Robinson [17, 19, 76, 77], and Moran et al [18, 78] by examining compartmental changes in hormone and cytokine expression following influenza virus infection. We linked seasonal influenza virus infection to clinical observations of adverse outcomes in pregnancy (preterm birth, SGA, stillbirth, increased maternal morbidity), enhanced lung and placental histopathology and reduced control of viral replication in lungs of infected pregnant mothers. We provide a model for how influenza virus infection, while contained in the lung, results in global dysregulation of the hormonal signaling required to sustain healthy gestation. In our study, the state of pregnancy dampens innate immune responses compared to non-pregnant controls by at least 50%; in some compartments, the maternal body maintains some form of systemic tolerance, while in the respiratory compartment, the lungs remain primed to fight invading pathogens.

Consistent with previous clinical reports and animal studies, we found that pregnancy increases the severity of seasonal influenza virus infection and thus impairs maternal and offspring health and recovery [25–27, 75, 79–81]. Pregnant mice exhibited increased viral replication in the lungs by 8-fold compared to infected, non-pregnant controls, a trend that was also found in similar studies looking at pandemic H1N1 influenza virus in 2012 and influenza B virus in 2014 [27, 75]. We found a significant correlation between lung viral load and progesterone levels in lung, placenta and serum suggesting that seasonal influenza virus infection during gestation disrupts the production of progesterone leading to preterm labor, SGA and increased fetal mortality. We also observed a dramatic increase of placental PGF2α after infection pointing to the role of this prostaglandin in the intense local inflammatory response. We also documented that the immune responses are compartment-specific in the mouse model, resulting in distinct signatures of inflammation within each compartment (S2 Fig). Similar to findings by Kim et al with pandemic 2009 H1N1 influenza virus, we demonstrated that seasonal H1N1 influenza virus enhanced lung pathology in pregnant mice via an increase in IL-6, IL-1α, and G-CSF expression and that infection reduced serum concentrations of progesterone during pregnancy [27]. The differences in systemic responses between pregnant and non-pregnant groups in our mouse model suggest that the unique endocrine environment supporting gestation compromises most of the innate immune responses to infection. These shifts in response to infection during pregnancy may have evolved to protect the fetus from activation of inappropriate activation of T cell responses, at the expense of increased inflammation in the lungs.

There are a few caveats to our pregnant mouse model of infection. Most obvious is that the average length of pregnancy in humans is 40 weeks while gestational length in mice is around 19–21 days depending on the mouse strain. However, both mice and humans possess a hemochorial placenta where the fetal chorion is in direct contact with maternal blood [82, 83]. Common transcription factors involving the expression of various placental genes have been identified in both human and mouse placenta [84]. Another caveat is the different mechanisms for maintaining progesterone secretions during gestation in mice and humans. In mice, progesterone is produced by the placental corpus luteum, which is regulated by prolactin for the first half of pregnancy and then by trophoblastic giant cells for the remainder of gestation [84]. In humans, prolactin is not required for the maintenance of pregnancy and the corpus luteum depends on the human chorionic gonadotropin (hCG) produced by trophoblasts to stimulate progesterone. After 8 weeks of gestation, the progesterone produced by the placenta is sufficient to maintain pregnancy in humans [84]. These differences should be taken into account when interpreting the role of progesterone in pregnancy following influenza virus infection. Lastly, Periolo et al. studied nasopharyngeal swab samples obtained in the second and third trimester of 41 pregnant women with confirmed pandemic H1N1 influenza A virus infection [2]. The authors reported increased expression of inflammatory cytokines IL-8, IL-6, and TNF-α and significantly lower levels of TGF-β and IFN-β compared to the pregnant women who survived or non-pregnant controls, with particular emphasis in the role of IL-6 in severity of disease. We have not seen these differences in seasonal H1N1 influenza virus-infected pregnant mouse model, and to our knowledge, there have been no studies examining the extent of cytokine expression in response to seasonal influenza virus infection in human pregnant women.

Influenza virus infection in our mouse model interfered with progesterone-mediated anti-inflammatory effects during pregnancy. Elevated levels of progesterone in the lungs during pregnancy suppress the activation of COX-2 by blocking IL-1β activation of NF-κB thus creating an anti-inflammatory environment at the expense of a quick response to foreign antigen [85]. However, when slow immune response results in uncontrolled viral growth, NF-κB initiates transcription of a host of immunomodulatory genes, including COX-2 [86]. It has been reported that influenza virus infection results in epithelial cell damage, which releases free oxygen radicals and activates COX-2 [42]. In our pregnant model we demonstrate that influenza virus infection induced COX-2 activation and upregulation of PGF2α in the lungs resulting in vasoconstriction and inflammation. These changes reduce the amount of oxygen available to the mother and developing fetus that may lead to respiratory distress, increased morbidity and retardation of fetal growth.

Previous studies have indicated that progesterone treatment at levels equivalent to hormonal birth control are sufficient to limit immunopathology of H1N1 influenza virus infection in non-pregnant mice [87, 88]. In our study, influenza A virus infection resulted in a decrease of progesterone expression in pregnant mice, and progesterone therapy may be a promising treatment to mitigate the effects of viral infection on lung pathology and to prevent PROM and early delivery of the fetus. COX-2 inhibitors have been proposed as antivirals for treating inflammation caused by influenza virus infection [89, 90]. This study demonstrates that pregnancy has a unique relationship with COX-2 upregulation in the lungs and that pregnant women may benefit from these molecular inhibitors following influenza virus infection.

Vertical transplacental transmission of influenza virus has been debated [29, 30, 91]. In our study, influenza virus was not detectable in the placenta and fetus. Instead, poor offspring health was likely due to imbalances in the maternal endocrine and immune physiology responsible for proper fetal development. Influenza virus infection resulted in a breakdown of the placental architecture, likely caused by inflammation, reduced progesterone expression and activation of structure-remodeling MMP-2 and MMP-9. Programmed cell death in amnion membranes is routine to normal parturition in rodents; membranes rupture in part due to tissue weakening rather than solely dependent on mechanical stress of labor [92]. Weakened strength of the amniotic membrane combined with reduced placental health creates an impetus for premature rupture of membranes (PROM) and thus, preterm birth. Increases in the placental concentrations of vasoconstrictor PGF2α and immune cellular activators G-CSF and RANTES following infection create an environment where uterine contractility is triggered prematurely and immune cells can be activated against fetal cells at the maternal-fetal interface (Figs 5B, S2). While we have shown disruption of key hormonal regulators in the lungs following infection during pregnancy, COX-2 does not seem to be involved in the phenotype of pre-term labor following viral infection during pregnancy. COX-2 is directly correlated with PGE2 levels, which do not change in the placenta following influenza virus infection. Rather, major changes in progesterone and PGF2α production have been shown to induce pre-term labor, while synthesis of PGF2α does not depend on COX-2 cellular expression [74]. Compromise of placental structure and function may lead to reduced oxygen and nutritional supply and buildup of gas and waste in the fetus, resulting in retardation of fetal growth and neural development or stillbirth. Clinical studies indicate that maternal influenza A virus infection during pregnancy may predispose offspring to psychosis and schizophrenia due to fetal neurodevelopment being dysregulated by hypoxia and inflammation [93, 94]. In future studies, offspring of influenza virus-infected mothers will be followed to adulthood and examined for neurological disorders as well as variation in the timing of viral dose at early, mid, and late gestation.

These data support a model where influenza virus infection “breaks through” the balance of maternal systemic tolerance towards developing fetuses, enhancing pathogenesis in the lungs and triggering pre-term birth thus affecting the health of both mother and offspring. We hypothesize that fetal health is impaired by the spillover of inflammatory cytokines, whose expression is influenced by pregnancy hormones, and structural remodeling proteins intended to repair maternal airways have long ranging effects outside the compartments they were intended to work in, such as the placenta. Previous studies have examined the effect of individual hormones on male and female innate immune responses to influenza virus infection. Klein et al have demonstrated that 17β-estradiol and progesterone treatments reduce influenza virus infection immunopathology, promoting expression of TGFβ, IL-22, and IL-6 and suppressing inflammatory cytokine production [77, 87, 95]. We found that reduced expression of progesterone in the lungs and placenta might be a causative factor for fetal and maternal respiratory distress and early termination of pregnancy.

Infection in the maternal lungs resulted in the upregulation of cellular activation markers in the placenta and inflammatory signaling in the fetus, indicating that while *in utero* offspring are not in contact with influenza virus; they are nevertheless negatively impacted by maternal infection. Our findings also suggest a breach of feto-placental tolerance, which relies on suppression of uterine and placental immune cells to ensure an inflammation-free environment for the developing fetus.

This study further explains the interconnected nature of hormonal and cytokine signaling during pregnancy and the unique predicament of maternal tolerance during respiratory viral infection. Recent work has shown that immunosuppression in the lungs during pregnancy creates an ideal environment for adaptation of influenza viruses to more virulent strains [81]. Thus, understanding how pregnancy hormones modulate immune responses to influenza virus infection is not just necessary for developing clinical interventions for a high-risk population but also as part of a global strategy to reduce the incidence of highly pathogenic influenza viruses with pandemic potential. Pregnant women are a target population for improving vaccination efficacy in order to reduce their risk of pregnancy complications due to infection and to increase protective strength of passive immunity to their offspring [96, 97]. We demonstrate that increased progesterone expression required to support pregnancy results in an immunosuppressive lung environment and that pregnancy increases susceptibility to prostaglandin-induced inflammation as a result of infection. These conclusions are important for understanding respiratory viral pathogenesis in a pregnant mother.

## Materials and methods

### Cells and virus stocks

Madin-Darby canine kidney (MDCK) cells (ATCC CCL 34, American Type Culture Collection, Manassas, VA) were maintained in Dulbecco's Modified Eagle's Medium (DMEM) (Mediatech, Herndon, VA) containing 10% fetal bovine serum (Hyclone, Thermo Scientific, Rockford, IL). A/Brisbane/59/07 (H1N1) virus stock was propagated in MDCK cells. The hemagglutination (HA) activity was determined using turkey blood cells (LAMPIRE Biological Laboratories, Pipersville, PA) [98]. The mouse-adapted A/Brisbane/59/07 (H1N1) strain was obtained by serially passaging the virus in lungs of BALB/c mice. The LD50 was determined by Reed-Munch formula [99] and the viral titers were determined by plaque assay [100]; 2xLD_50_ mouse-adapted virus is approximately 155 plaque forming units (p.f.u) per infection.

### Animals

8-week-old female BALB/c mice (Harlan Laboratories, Dublin, VA) were bred and housed in a biosafety level 1 facility for breeding; infections were conducted in a biosafety level 2 at Emory University Whitehead animal facility. All animal studies were approved by the IACUC at Emory University.

### Protocol for timed pregnancies

The mouse estrous cycle is divided into four stages: estrous, metestrous, diestrous, and proestrous. Each stage can be determined by visual examination of vaginal opening [101]. Tagged female mice were used for breeding. Cages were set up with three to four female mice in proestrous or estrus and one a male for 3 days [102]. Females were monitored for the presence of a copulation plug, indicative of mating and body weight changes were monitored daily. In uninfected BALB/c pregnant mice, gestation lasts approximately 21 days, and pregnancy can be determined when mice gain 20% of their initial body weight. Pregnant mice were placed in separate cages, two females per cage, and the remaining mice were mated again.

### Animal infection with seasonal influenza virus and sample collection

Pregnant females that had noticeable body weight increases (in the range of 20–25%) of their initial bodyweights between days 12–14 after mating were infected with 2xLD_50_ mouse-adapted A/Brisbane/59/07 (dose calculated for non-pregnant, healthy 8-week-old female mice). Intranasal infections were performed under light isoflurane anesthetization, and euthanasia was performed via CO_2_ asphyxiation. Severity of infection, completion of gestation, and health outcome of offspring (i.e. healthy, small for gestational age, or stillborn) were all closely monitored and compared to uninfected pregnant mice.

In another cohort of mice, sera, lungs, placenta, and fetuses were collected 4 d.p.i. in order to assess viral presence and extend of inflammation in various compartments, host innate immune responses, and hormone expression. Serum was stored at -20°C in the presence of Halt Protease Inhibitor (Thermo Fisher) until further use.

### PCR

Lung and placental lysates were harvested from infected and uninfected pregnant mice and homogenized, filtered through 70μm filters and stored in 1x DMEM, 1x Halt Protease Inhibitor and/or RNAse Later (Ambion). Viral RNA was isolated using a QIAamp Viral RNA Mini Kit (Qiagen) and probed for the presence of H1N1 HA and M genes via qPCR. Viral RNA was converted to cDNA iTaq Universal SYBR Green Supermix (Bio-Rad) and amplified using 3 nmol of commercially available or synthesized primers in a CFX96 Real-Time PCR Detection System (Bio-Rad). PCR mixtures were heated at 50°C for 10 minutes to convert RNA to cDNA, 95°C for 1 minute, and cycled 40x (95°C for 10 seconds, annealing temperatures were varied dependent on primer melting curve for 15 seconds, and 72°C for 30 seconds). A melting curve from 65°C to 95°C was performed to assess quality of primer binding. H1 HA was amplified using Influenza A Virus H1 Primers (BEI Resources, NR-12316, with an annealing temperature of 61°C. H1 M was amplified using synthesized primers (FLUAM-1F: AAGACCAATCCTGTCACCTCTGA; FLUAM-1R: CAAAGCGTCTACGCTGCAGTCC; Operon) with an annealing temperature of 61°C, and mouse GAPDH (realtimeprimers.com, VMPS-7317) with an annealing temperature of 50°C [103]. Analysis of threshold values and normalization to GAPDH were performed in CFX Manager Software (Bio-Rad). PCR products were electrophoresed on a 1% agarose/TAE gel and imaged using ethidium bromide.

### Plaque assays

Viral titers in the lung tissue samples were quantified via plaque assay in MDCK cells as described previously [104]. Viral titers were assessed per gram of tissue.

### Determination of hormone levels

Progesterone, prostaglandin F2α (PGF2α) and prostaglandin E2 (PGE2) were quantified via ELISA kits from ALPCO (Salem, NH). Experimental values for lungs and placentas were normalized by the mass of the tissue and the volume in which samples were homogenized.

### Cytokine expression

For cytokine expression, a 23-plex Luminex assay from Bio-Rad (Hercules, CA) was used. Experimental values for lungs, placentas, and fetuses were normalized by volume and mass of the tissues. Fold changes were expressed relative to uninfected controls. A heat map was constructed to visualize fold changes within the tissues in pregnant and non-pregnant mice.

### MMP, COX-2, and PIBF quantification in tissue lysates

Molecular analysis of protein expression was performed on 5 mg of placental tissue lysate via Western blot. Purified anti-murine MMP-9 (BioLegend 819701, 1:500) and anti-murine MMP-2 (BioLegend 680002, 1:500) were used to detect degradative proteins and secondary antibodies (goat anti-mouse, 1:10,000). Rabbit anti-murine COX-2 (Abcam, ab52237) and rabbit anti-murine C13orf24 (PIBF) (Abcam, ab156267) were used to detect markers for oxidative and hormonal stress in the lungs and placenta, and goat anti-rabbit (1:10,000) antibodies were used as secondary antibodies. Rabbit anti-murine β-actin (1:2000) was used as a loading control with secondary antibodies (goat anti-rabbit, 1:10,000). Blots were developed with Super Signal Femto Maximum Sensitivity Substrate (Thermo Scientific 34096) and imaged using a Bio-Rad ChemiDoc Touch. Volume intensity of MMP-2 and MMP-9 was normalized to β-actin loading controls. A two-way ANOVA analyzing factors of protein maturation (p<0.0001, ***) and infection (P<0.0296, *) was performed in GraphPad Prism 7.

### Histology

Placentas were isolated 4 d.p.i. (day 16 of gestation) and submerged in histology cassettes in 4% paraformaldehyde overnight at 4°C. Tissues were embedded in paraffin, sectioned in 4 μm, and fixed to glass microscopy slides. Hematoxylin and eosin staining was performed by Yerkes National Primate Research Center Pathology Core, and slides were imaged on a Zeiss Akioskop with SpotFlex 15.2 camera with Spot Advanced 4.7 software. Pathology scores were determined by randomizing histology slides prior to imaging and blindly scoring the incidence of exposed fetal endothelial nuclei (FEN), degradation in the spongiotrophoblast layer, and fibrinoid necrosis (FN).

### Statistics

All statistical analysis used a student’s t-test, linear regression, or one-way ANOVA using GraphPad Prism statistical software with a significance level (α) of 0.05.

### Ethics statement

Emory University Division of Animal Resources veterinary staff ascertained welfare of animals in addition to research scientists, and DAR staff performed regular care and wellness assessments. Animal work was conducted according to Emory University Institutional Animal Care and Use Committee (IACUC) guidelines according to an approved protocol (DAR2002950-122617BN) in accordance with the United States federal Animal Welfare Act (PL 89–544) and subsequent amendments. Emory University is registered with the United States Department of Agriculture (57-R-003) and has filed an Assurance of Compliance statement with the Office of Laboratory Animal Welfare of the National Institutes of Health (D16-00113). Emory University has been fully and continuously accredited by AAALAC International since 1992 (Unit 000781). The Georgia Fee-Exempt Wild Animal Permit Customer Number for animals maintained by the Division of Animal Resources is 22257.

### Accession numbers

The sequence for each genomic segment of the mouse-adapted virus used in this study may be found at NCBI GenBank MG460793 (HA), MG460794 (M1), MG460795 (NA), MG460796 (NP), MG460797 (NS1), MG460798 (PA), MG460799 (PB1), MG460800 (PB2).

## Supporting information

S1 Fig Viral titer in the lungs affects expression of progesterone and PGF2α in a compartment-specific manner. Hormone expression was quantified in sera, lung and placental lysates in pregnant infected mice with ELISA, and viral load was quantified with plaque assay in MDCK-derived cells. Virus titer detected in the lungs was plotted against corresponding expression values; linear regression (blue line) was derived for each compartment and 95% confidence is represented by dashed lines. Serum PGF2α was correlated with lung viral titer (A, r^2^ = 0.4) and lungs (B, r^2^ = 0.6) of pregnant mice, and progesterone was correlated with lung viral titer in serum (C, r^2^ = 0.8), lung lysates (D, r^2^ = 0.7), and placenta (E, r^2^ = 0.46).
(TIF)

S2 Fig Infection and pregnancy impact cytokine expression in a compartment-specific manner. Cytokine and chemokine expression in serum, lungs, placenta and fetuses was determined via Bio-Rad 23-plex Luminex Assay. The effect of infection on cytokine and chemokine expression is represented as fold-change of the average values of infected mice over average values of uninfected mice. Numerical values and fold changes are reported in Supplementary S1–S3 Tables.
(TIF)

S1 Table Serum chemokine and cytokine levels 4 days post-infection. Serum was collected from pregnant and non-pregnant infected and non-infected mice 4 d.pi. (16 days post mating), and cytokine and chemokine expression was quantified via Bio-Rad 23-plex Luminex Assay. The shaded fold-differences are significant (p<0.05). * RANTES is the only chemokine that is significantly decreased after infection in serum of pregnant mice. *P* values were determined with t-test comparing infected and uninfected tissues (n = 5–14).
(DOCX)

S2 Table Lung chemokine and cytokine levels 4 days post-infection. Lungs were collected from pregnant and non-pregnant infected and non-infected mice 4 d.p.i. (E16 gestation), and cytokine and chemokine expression was quantified in homogenized lysates via Bio-Rad 23-plex Luminex Assay. The shaded fold-differences are significant (p≤0.05). *P* values were determined with t-test comparing infected and uninfected tissues (n = 5–14).
(DOCX)

S3 Table Placental and fetal chemokine and cytokine levels 4 days post-infection. Placentae and fetuses were collected from pregnant and non-pregnant infected and non-infected mice 4 d.p.i. (E16 gestation), and cytokine and chemokine expression was quantified in homogenized lysates via Bio-Rad 23-plex Luminex Assay. The shaded fold-differences are significant (p≤0.05). *: depicts increases after infection. G-CSF and RANTES are increased in placenta and IL-1α is increased in fetus after infection. *P* values were determined with t-test comparing infected and uninfected tissues (n = 5–14).
(DOCX)
