# Supplementary figures and images for: H1N1 influenza virus infection results in adverse pregnancy outcomes by disrupting tissue-specific hormonal regulation
Source: PLoS Pathog. 2017 Nov 27;13(11):e1006757. doi: 10.1371/journal.ppat.1006757 (PMC5720832; doi:10.1371/journal.ppat.1006757)

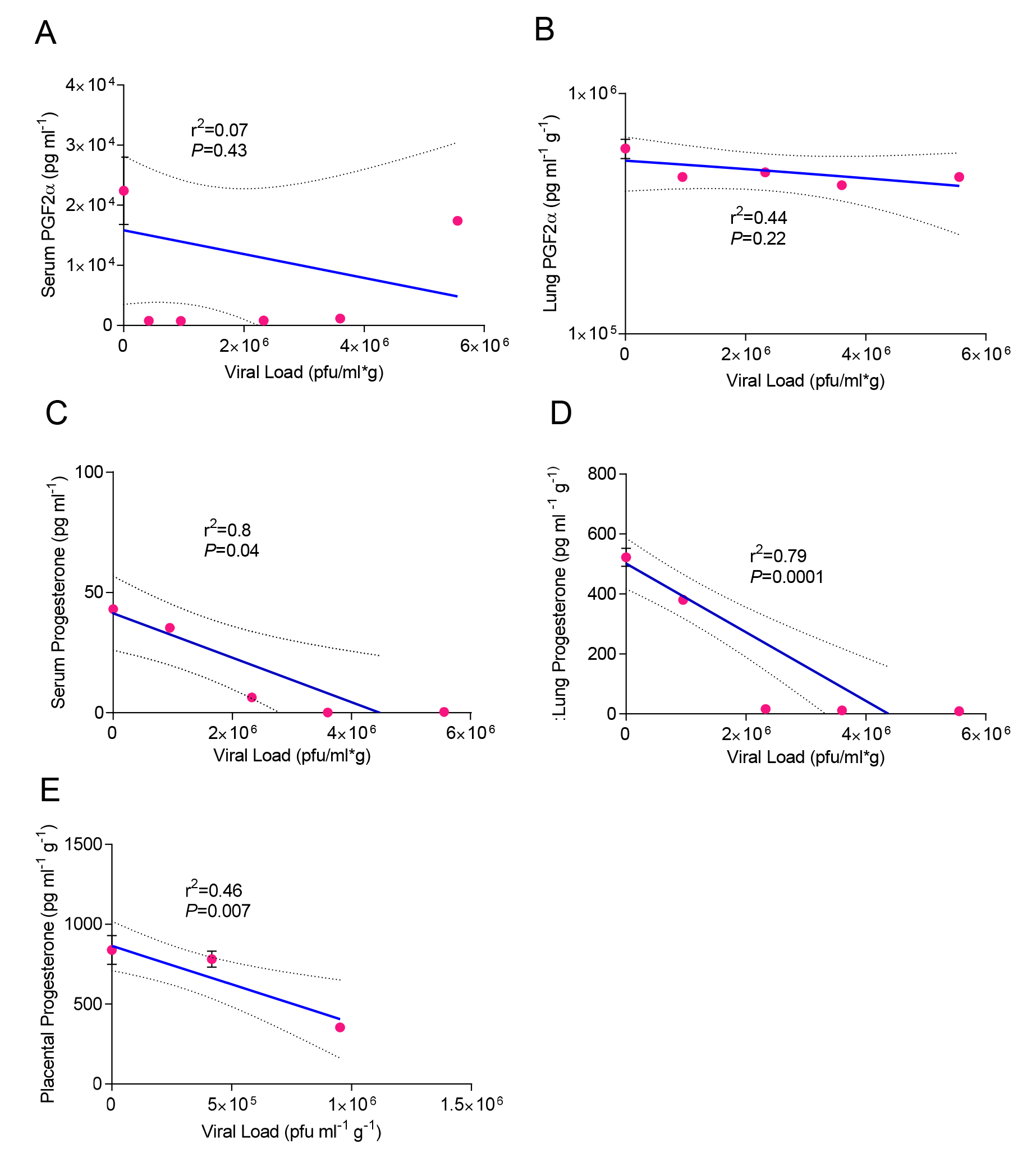

Supplement: S1 Fig — Hormone expression was quantified in sera, lung and placental lysates in pregnant infected mice with ELISA, and viral load was quantified with plaque assay in MDCK-derived cells. Virus titer detected in the lungs was plotted against corresponding expression values; linear regression (blue line) was derived for each compartment and 95% confidence is represented by dashed lines. Serum PGF2α was correlated with lung viral titer (A, r2 = 0.4) and lungs (B, r2 = 0.6) of pregnant mice, and progesterone was correlated with lung viral titer in serum (C, r2 = 0.8), lung lysates (D, r2 = 0.7), and placenta (E, r2 = 0.46). (TIF) [file ppat.1006757.s001.tif]

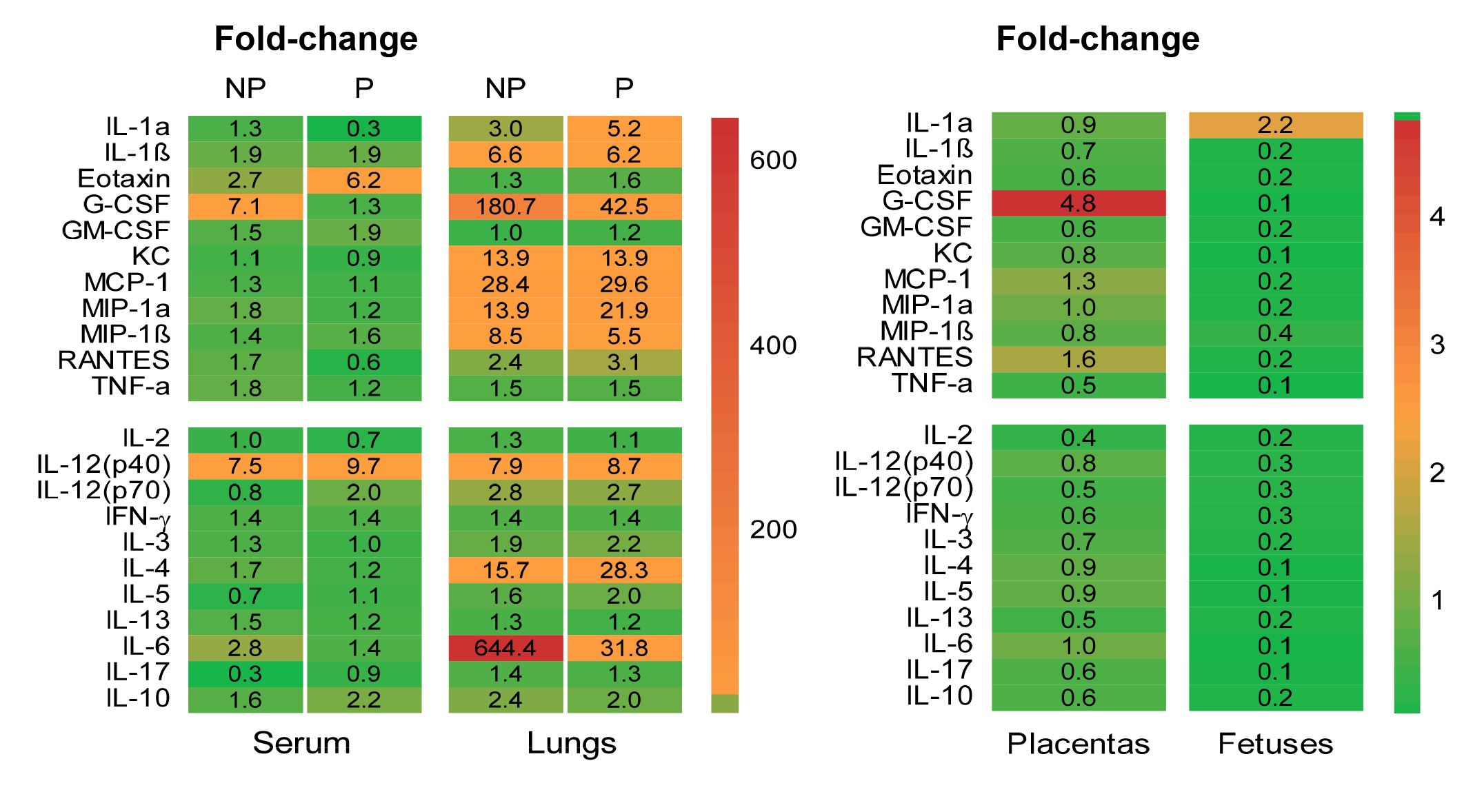

Supplement: S2 Fig — Cytokine and chemokine expression in serum, lungs, placenta and fetuses was determined via Bio-Rad 23-plex Luminex Assay. The effect of infection on cytokine and chemokine expression is represented as fold-change of the average values of infected mice over average values of uninfected mice. Numerical values and fold changes are reported in Supplementary S1–S3 Tables. (TIF) [file ppat.1006757.s002.tif]
