# Supplementary material for: H1N1 influenza virus infection results in adverse pregnancy outcomes by disrupting tissue-specific hormonal regulation
Source: PLoS Pathog. 2017 Nov 27;13(11):e1006757. doi: 10.1371/journal.ppat.1006757 (PMC5720832; doi:10.1371/journal.ppat.1006757)
Supplement: S1 Table — Serum was collected from pregnant and non-pregnant infected and non-infected mice 4 d.pi. (16 days post mating), and cytokine and chemokine expression was quantified via Bio-Rad 23-plex Luminex Assay. The shaded fold-differences are significant (p<0.05). * RANTES is the only chemokine that is significantly decreased after infection in serum of pregnant mice. P values were determined with t-test comparing infected and uninfected tissues (n = 5–14). (DOCX) [file ppat.1006757.s003.docx]

|  | | | | | |  |  |  |
| --- | --- | --- | --- | --- | --- | --- | --- | --- |
|  | **Pregnant** | | | | **Non-Pregnant** | | | |
| **Cytokine** | **Non-Infected** | **Infected** | **Fold** | ***P*** | **Non-Infected** | **Infected** | **Fold** | ***P*** |
| **IL-1****α** | 47.2 ±28.8 | 14.6 ±4.6 | 0.3 | 0.06 | 53.7 ± 51.7 | 67.1 ±38.8 | 1.3 | 0.66 |
| **IL-1β** | 122.4 ±38.2 | 230.7 ±42.5 | 1.9 | 0.01 | 207.6 ±48.8 | 383 ±175.9 | 1.8 | 0.00 |
| **Eotaxin** | 528.0 ±219.0 | 3254.6 ±932.5 | 6.2 | 0.00 | 823.0 ±420.9 | 2245.7 ±952.2 | 2.7 | 0.01 |
| **G-CSF** | 150.8 ±92.7 | 192.6 ±115.7 | 1.3 | 0.55 | 57.9 ±53.1 | 409.7 ±194.7 | 7.1 | 0.00 |
| **GM-CSF** | 99.7 ±55.0 | 187 ±35.5 | 1.9 | 0.02 | 197.9 ±27.1 | 298.6 ±117.1 | 1.5 | 0.01 |
| **KC** | 23.9 ±5.1 | 20.3 ±7.0 | 0.9 | 0.39 | 36.3 ±18.2 | 39.6 ±22.4 | 1.1 | 0.81 |
| **MCP-1** | 216.2 ±41.2 | 238.3 ±68.1 | 1.1 | 0.55 | 225.7 ±71.3 | 288.6 ±105.8 | 1.3 | 0.27 |
| **MIP-1α** | 15.5 ±3.1 | 18.7 ±8.6 | 1.2 | 0.46 | 15.6 ±12.3 | 28.6 ±8.7 | 1.8 | 0.00 |
| **MIP-1β** | 41.4 ±7.3 | 66.9 ±23.5 | 1.6 | 0.07 | 39.9 ±16.0 | 53.8 ±18.1 | 1.3 | 0.26 |
| **RANTES** | 57.4 ±16.0 | 31.3 ±15.3 | 0.5* | 0.03 | 78.6 ±18.5 | 131.4 ±38.0 | 1.7 | 0.00 |
| **TNF-α** | 456.8 ±122.5 | 565.7 ±188.4 | 1.2 | 0.32 | 385.4 ±111.8 | 702.6 ±198.9 | 1.8 | 0.00 |
| **IL-12p40** | 91.7 ±24.3 | 891.9 ±209.4 | 9.7 | 0.00 | 249.3 ±39.7 | 1856.7 ±945.5 | 7.4 | 0.00 |
| **IL-12p70** | 238.2 ±63.7 | 478.4 ±295.5 | 2.0 | 0.14 | 175.1 ±57.6 | 135.6 ±89.7 | 0.8 | 0.43 |
| **IL-6** | 10.4 ±4.8 | 14.3 ±4.6 | 1.4 | 0.23 | 10.5 ±3.1 | 29.7 ±8.9 | 2.8 | 0.00 |
| **IL-17** | 188.3 ±54.9 | 170.1 ±93.9 | 0.9 | 0.72 | 81.3 ±14.8 | 21.3 ±60.7 | 0.3 | 0.00 |
| **IL-2** | 27.6 ±5.5 | 18.7 ±7.3 | 0.7 | 0.06 | 36.9 ±17.1 | 35.9 ±8.9 | 1.0 | 0.91 |
| **IFN-γ** | 24.2 ±10.1 | 33.2 ±22.0 | 1.4 | 0.44 | 25.6 ±8.3 | 35.5 ±8.6 | 1.4 | 0.05 |
| **IL-3** | 11.2 ±5.4 | 11.2 ±3.7 | 1.0 | 0.99 | 9.4 ±3.4 | 12.5 ±5.7 | 1.3 | 0.32 |
| **IL-4** | 8.8 ±1.0 | 10.3 ±3.2 | 1.2 | 0.36 | 9.6 ±2.3 | 16.4 ±4.8 | 1.7 | 0.00 |
| **IL-5** | 10.6 ±2.8 | 11.5 ±3.1 | 1.1 | 0.64 | 22.2 ±11.9 | 14.8 ±6.2 | 0.7 | 0.26 |
| **IL-13** | 244.9 ±79.6 | 303.8 ±114.1 | 1.2 | 0.38 | 300.3 ±97.4 | 444.3 ±122.7 | 1.5 | 0.03 |
| **IL-10** | 18.9 ±13.7 | 42.3 ±12.2 | 2.2 | 0.02 | 28.5±19.7 | 45.6 ±48.5 | 1.6 | 0.48 |
