# Supplementary material for: H1N1 influenza virus infection results in adverse pregnancy outcomes by disrupting tissue-specific hormonal regulation
Source: PLoS Pathog. 2017 Nov 27;13(11):e1006757. doi: 10.1371/journal.ppat.1006757 (PMC5720832; doi:10.1371/journal.ppat.1006757)
Supplement: S2 Table — Lungs were collected from pregnant and non-pregnant infected and non-infected mice 4 d.p.i. (E16 gestation), and cytokine and chemokine expression was quantified in homogenized lysates via Bio-Rad 23-plex Luminex Assay. The shaded fold-differences are significant (p≤0.05). P values were determined with t-test comparing infected and uninfected tissues (n = 5–14). (DOCX) [file ppat.1006757.s004.docx]

|  | | | | | |  |  |  |
| --- | --- | --- | --- | --- | --- | --- | --- | --- |
|  | **Pregnant** | | | | **Non-Pregnant** | | | |
|  | **Non-Infected** | **Infected** | **Fold** | ***P*** | **Non-Infected** | **Infected** | **Fold** | ***P*** |
| **IL-1α** | 81 ±24.8 | 418.2 ±89.7 | 5.2 | 0.00 | 85 ±23.6 | 259.8 ±83.0 | 3.0 | 0.01 |
| **IL-1β** | 686 ±195.5 | 4238.9 ±303.5 | 6.2 | 0.00 | 416 ±116.8 | 2745.1 ±991.2 | 6.6 | 0.01 |
| **Eotaxin** | 6074 ±1861.1 | 9713.5 ±428.8 | 1.6 | 0.00 | 7492 ±2319.3 | 9414.2 ±2172.2 | 1.3 | 0.19 |
| **G-CSF** | 16 ±12.3 | 1095.8 ±1290.7 | 42.5 | 0.03 | 6 ±1.6 | 1020.1 ±587.6 | 180.7 | 0.02 |
| **GM-CSF** | 278 ±83.4 | 330.3 ±24.1 | 1.2 | 0.05 | 345 ±106.9 | 345.8 ±81.9 | 1.0 | 0.99 |
| **KC** | 665 ±194.6 | 9240.2 ±1210.3 | 13.9 | 0.00 | 483 ±143.3 | 6710.3 ±2062.1 | 13.9 | 0.00 |
| **MCP-1** | 915 ±339.6 | 27077.3 ±5265.3 | 29.6 | 0.00 | 745 ±296.5 | 21165.3 ±9307.2 | 28.4 | 0.01 |
| **MIP-1a** | 140 ±57.4 | 3062.7 ±572.2 | 21.9 | 0.00 | 233 ±168.7 | 3242.1 ±866.4 | 13.9 | 0.00 |
| **MIP-1β** | 166 ±46.6 | 920.2 ±257.0 | 5.5 | 0.00 | 177 ±96.8 | 1505 ±352.7 | 8.5 | 0.00 |
| **RANTES** | 3477 ±1936.2 | 10886.7 ±2398.6 | 3.1 | 0.00 | 5365 ±2721.6 | 12644.6 ±7420.3 | 2.4 | 0.39 |
| **TNF-α** | 1185 ±355.7 | 1764 ±478.0 | 1.5 | 0.05 | 1050 ±298.1 | 1593.12 ±381.0 | 1.5 | 0.03 |
| **IL-12p40** | 564 ±560.4 | 4879.9 ±2045.1 | 8.7 | 0.01 | 634 ±625.4 | 5021.8 ±2050.2 | 7.9 | 0.01 |
| **IL-12p70** | 201 ±53.5 | 534.5 ±46.1 | 2.7 | 0.00 | 208 ±77.7 | 578 ±145.3 | 2.8 | 0.00 |
| **IL-6** | 9 ±9.5 | 286.2 ±102.3 | 31.8 | 0.01 | 1.17 ±2.65 | 754.1 ±204.5 | 644.4 | 0.00 |
| **IL-17** | 38 ±6.0 | 50.8 ±7.8 | 1.3 | 0.02 | 35 ±7.4 | 49.9 ±9.2 | 1.4 | 0.02 |
| **IL-2** | 64 ±9.5 | 69.7 ±17.7 | 1.1 | 0.51 | 81 ±42.4 | 104.5 ±41.9 | 1.3 | 0.38 |
| **IFN-γ** | 47 ±9.3 | 65 ±12.1 | 1.4 | 0.03 | 54 ±18.9 | 77.9 ±9.4 | 1.4 | 0.03 |
| **IL-3** | 36 ±9.6 | 77.2 ±19.8 | 2.2 | 0.01 | 41 ±14.9 | 77.3 ±13.1 | 1.9 | 0.00 |
| **IL-4** | 2 ±5.2 | 68.6 ±20.1 | 28.3 | 0.00 | 5 ±11.3 | 72.3 ±22.9 | 15.7 | 0.00 |
| **IL-5** | 38 ±19.4 | 73.4 ±43.1 | 2.0 | 0.14 | 34 ±14.4 | 55.7 ±30.8 | 1.6 | 0.21 |
| **IL-13** | 796 ±286.1 | 920.5 ±72.9 | 1.2 | 0.15 | 715 ±152.4 | 895.3 ±205.4 | 1.3 | 0.15 |
| **IL-10** | 110 ±22.3 | 218.4 ±21.2 | 2.0 | 0.00 | 100 ±30.3 | 234.5 ±46.6 | 2.4 | 0.00 |
