# Supplementary material for: H1N1 influenza virus infection results in adverse pregnancy outcomes by disrupting tissue-specific hormonal regulation
Source: PLoS Pathog. 2017 Nov 27;13(11):e1006757. doi: 10.1371/journal.ppat.1006757 (PMC5720832; doi:10.1371/journal.ppat.1006757)
Supplement: S3 Table — Placentae and fetuses were collected from pregnant and non-pregnant infected and non-infected mice 4 d.p.i. (E16 gestation), and cytokine and chemokine expression was quantified in homogenized lysates via Bio-Rad 23-plex Luminex Assay. The shaded fold-differences are significant (p≤0.05). *: depicts increases after infection. G-CSF and RANTES are increased in placenta and IL-1α is increased in fetus after infection. P values were determined with t-test comparing infected and uninfected tissues (n = 5–14). (DOCX) [file ppat.1006757.s005.docx]

|  | | | | | |  |  |  |
| --- | --- | --- | --- | --- | --- | --- | --- | --- |
|  | **Placentas** | | | | **Fetuses** | | | |
|  | **Non-Infected** | **Infected** | **Fold** | ***P*** | **Non-Infected** | **Infected** | **Fold** | ***P*** |
| **IL-1α** | 5299.6 ±3340.4 | 4710.7 ±1390.0 | 0.9 | 0.53 | 114.7 ±164.3 | 248.5 ±210.7 | 2.2* | 0.05 |
| **IL-1β** | 10948.2 ± 3540.3 | 7319.6 ±1032.6 | 0.7 | 0.00 | 477.8 ±371.8 | 95.5 ±46.3 | 0.2 | 0.00 |
| **Eotaxin** | 93402.3 ±29970.0 | 57049.4 ±14303.5 | 0.6 | 0.00 | 9701.6 ±8126.5 | 1561.9 ±527.6 | 0.2 | 0.00 |
| **G-CSF** | 7918.5 ±4603.7 | 38170.7 ±31120.6 | 4.8* | 0.01 | 170.4 ±177.4 | 27.5 ±11.2 | 0.1 | 0.07 |
| **GM-CSF** | 4868.3 ±1507.5 | 2846.5 ±698.9 | 0.6 | 0.00 | 260.5 ±222.5 | 43.2 ±14.4 | 0.2 | 0.00 |
| **KC** | 12712.5 ±6675.8 | 10225.3 ±6773.7 | 0.8 | 0.33 | 331.7 ±538.8 | 41.8 ±16.2 | 0.1 | 0.04 |
| **MCP-1** | 15782.3 ± 7165.0 | 21031.3 ±8454.5 | 1.3 | 0.09 | 761 ±754.0 | 141.4 ±45.4 | 0.2 | 0.01 |
| **MIP-1α** | 3229.6 ±2436.6 | 3311.3 ±1045.0 | 1.0 | 0.91 | 126.6 ±126.8 | 20 ±5.3 | 0.2 | 0.00 |
| **MIP-1β** | 1073.6 ±419.1 | 889.8 ±271.4 | 0.8 | 0.17 | 70.8 ±71.3 | 26.4 ±11.1 | 0.4 | 0.03 |
| **RANTES** | 871.7 ±347.8 | 1347.3 ±409.6 | 1.5* | 0.00 | 63.7 ±92.7 | 10.4 ±3.7 | 0.2 | 0.04 |
| **TNF-α** | 14045.4 ±3807.7 | 6884.2 ±1370.9 | 0.5 | 0.00 | 859.7 ±597.5 | 113.9 ±54.2 | 0.1 | 0.00 |
| **IL-12p40** | 2486.6 ±823.7 | 2026.2 ±377.2 | 0.8 | 0.06 | 62.9 ±50.8 | 18.6 ±4.4 | 0.3 | 0.00 |
| **IL-12p70** | 4853.4 ±119.8 | 2620.9 ±603.7 | 0.5 | 0.00 | 252.2 ±221.5 | 73.7 ±15.3 | 0.3 | 0.01 |
| **IL-6** | 2105.2 ±987.4 | 2085.6 ±570.4 | 1.0 | 0.95 | 111 ±109.4 | 13.1 ±7.6 | 0.1 | 0.02 |
| **IL-17** | 462.7 ±88.0 | 297.8 ±82.6 | 0.6 | 0.00 | 37.6 ±29.1 | 4.9 ±2.4 | 0.1 | 0.00 |
| **IL-2** | 1868.9 ±472.1 | 701.4 ±175.1 | 0.4 | 0.00 | 106.1 ±71.8 | 25 ±12.5 | 0.2 | 0.00 |
| **IFN-γ** | 1327.7 ±378.1 | 795.3 ±211.2 | 0.6 | 0.00 | 140.3 ±132.7 | 38 ±13.2 | 0.3 | 0.01 |
| **IL-3** | 291.8 ±78.7 | 206.9 ±52.4 | 0.7 | 0.00 | 10.8 ±9.6 | 2.5 ±1.3 | 0.2 | 0.01 |
| **IL-4** | 434.1 ±111.7 | 373.3 ±117.5 | 0.9 | 0.17 | 90.6 ±16.4 | 9.8 ±4.5 | 0.1 | 0.09 |
| **IL-5** | 384.8 ±155 | 330.5 ±183.8 | 0.9 | 0.40 | 67.9 ±51.2 | 8.6 ±4.6 | 0.1 | 0.00 |
| **IL-13** | 24155.9 ±6693.4 | 12923.9 ±2948.0 | 0.5 | 0.00 | 917.6 ±768.7 | 177.7 ±50.7 | 0.2 | 0.00 |
| **IL-10** | 1402.6 ±418.5 | 894.7 ±212.9 | 0.6 | 0.00 | 53.3 ±47.4 | 10.2 ±3.5 | 0.2 | 0.00 |
